# Exploring MXene Materials in Energy Storage Devices: A Review of Supercapacitor Applications

**DOI:** 10.1021/acsmaterialsau.5c00102

**Published:** 2025-08-21

**Authors:** Lucas de Sousa Silva, Eudes Eterno Fileti, Guilherme Colherinhas

**Affiliations:** † Instituto de Física, 67824 Universidade Federal de Goiás. 74690-900 Goiânia, Goiás, Brazil; ‡ Instituto de Ciência e Tecnologia, 28105 Universidade Federal de São Paulo, São José dos Campos, 12247-014 São Paulo, Brazil

**Keywords:** energy storage, supercapacitors, MXene, materials, transition metal, synthesis, experimental, computational, electrodes, electrochemical performance

## Abstract

The pursuit of advancements in energy storage is critical to making human activities more efficient and practical. Supercapacitors (SCs) are a promising alternative, offering high power density and long cycle life. The efficiency of these devices largely depends on the careful selection of materials for their electrodes and electrolytes. MXene, an emerging class of two-dimensional materials composed of transition metal carbides and nitrides, have shown significant potential as electrodes for energy storage devices. This review covers MXene electrodes in supercapacitors, integrating computational and experimental results. Based on the data from the reviewed literature, computational studies indicate capacitance values ranging from 8.19 μF/cm^2^ to 3293.00 μF/cm^2^ and from 252.2 F/g to 291.5 F/g. Experimental studies, in turn, report capacitance values from 26 F/g to 556 F/g and voltage windows reaching up to 1.4 V. The study explores their structural and electrical properties and their applicability in high-performance devices. Finally, we discuss the challenges in MXene research, highlighting current difficulties and providing insights into opportunities and future directions for developing more efficient energy storage solutions.

## Graphical abstract

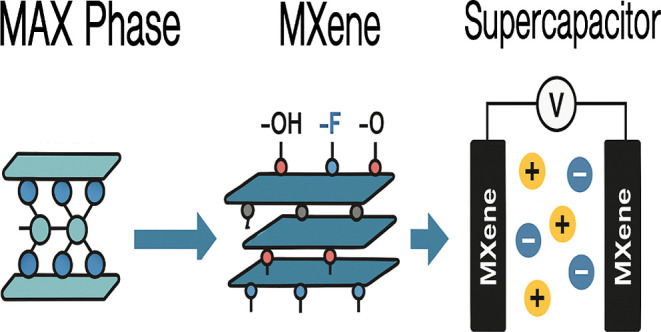

## 1 Introduction

Since the Industrial Revolution, energy has been the driving force behind human progress, fueling technological advancements and economic growth. From coal to oil and, more recently, to renewable sources, the development of society has always been closely tied to the ability to store and use energy efficiently.^1^ Today, the quest for innovative energy storage technologies has become essential.^2,3^ In this context, batteries and supercapacitors (SCs) have emerged as promising solutions for energy storage. Batteries operate by exploiting redox reactions that occur in their electrodes, primarily involving electron transfer to convert chemical energy into electrical energy.^4–6^

SCs consist of two oppositely charged electrodes separated by an electrolyte and are classified as electric double layer capacitors (EDLCs), pseudocapacitors or hybrids.^7^ In EDLCs, charge storage occurs through the formation of an electric double layer (EDL) during the charging process, driven by the accumulation of charges at the electrode–electrolyte interface without charge transfer between the two.^7–10^ In contrast, pseudocapacitors store charge via redox reactions, similar to batteries, involving charge transfer between the electrode and the electrolyte.^7^ Hybrid supercapacitors, conversely, combine the properties of EDLCs and pseudocapacitors, leveraging the advantages of both systems for charge storage.^7^

In both devices, selecting appropriate materials for the electrodes and electrolyte is crucial to enhance efficiency. Since the discovery of graphene in 2004,^11^ various other materials have been studied to assess their feasibility as electrodes in energy storage devices. The discovery of Ti_3_C_2_ in 2011 ^12^ marked the emergence of a new class of materials known as MXene. This breakthrough was achieved when Naguib and his collaborators^12^ successfully removed aluminum layers from the Ti_3_AlC_2_ compound using hydrofluoric acid (HF). MXene are a class of two-dimensional materials synthesized from MAX phases, which are ternary carbides or nitrides with the general formula M_*n*+1_AX_*n*_, where M represents a transition metal (e.g., Sc, Ti, V, Cr, Zr, Hf, Nb, Mo, Ta, and W), A is an element from group 13 or 14 (such as Al or Si), and X stands for carbon or nitrogen.^12^ Through the synthesis process, the A element is selectively removed via chemical etching, leading to the formation of MXene, which have the general formula M_*n*+1_X_*n*_T_*x*_ (*n* = 1, 2, 3). In this formula, T_*x*_ denotes surface functional groups, such as –O, –OH, –F, or even −Cl, which are introduced during the synthesis and play a crucial role in determining the physical and chemical properties of these materials. A schematic model of the structures of the MAX phase and MXene can be seen in Figure 1.

**Figure 1:**
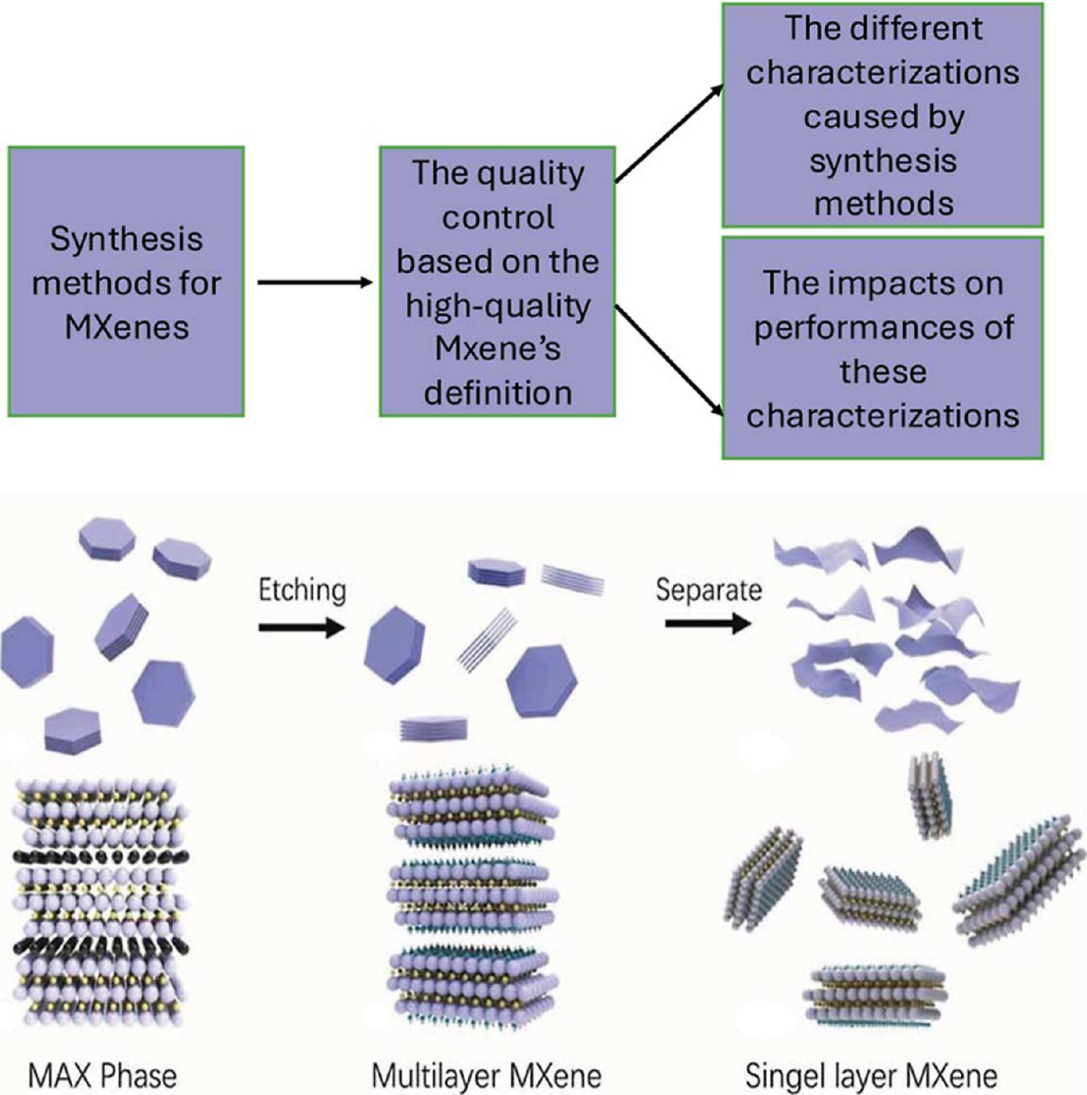
Consecutive steps of schematic steps for MXene synthesis from their MAX phase, taking Ti_3_AlC_2_ for the precursor. Adapted with permission under a Creative Commons (CC BY 4.0), from ref 13. Copyright 2021 Taylor & Francis Group.

MXene offer several advantages in the energy storage field: (1) they exhibit high electronic conductivity, facilitating electron transfer; (2) their single-layer structure provides a low-energy barrier for rapid ion diffusion; (3) they feature interlayer spaces capable of accommodating charges during charge–discharge cycles; (4) their surface functional groups make MXene highly suitable for building strong and efficient connections with other materials for ion-metal storage applications.^14,15^ In addition, MXene exhibit a broad diversity of chemical compositions due to the wide range of available M elements.^16,17^ Furthermore, a significant advantage of this class of materials is the ability to employ various preparation methods to modulate the functional groups on their surface terminations.^16,17^ Another noteworthy feature is the presence of hydrogen bonds on their surface terminations, which enhances the hydrophilicity of MXene.^16,17^ This increased interaction with water molecules is particularly advantageous from an energy storage perspective. Studies on the hydration of electrolytes have shown that water can significantly enhance the energy density of these devices.^18–20^

Given the importance, this review aims to provide a comprehensive analysis of MXene, highlighting the key synthesis and production methods, with a particular emphasis on techniques that enable the modulation of surface terminations to enhance their functional properties. Additionally, the most relevant results from the literature will be presented and critically discussed, evaluating the performance of these materials across various energy storage contexts. Finally, the review will explore innovative methods and future perspectives to optimize the properties of MXene, further expanding their applicability as electrodes in high-performance devices. This review covers a broad range of MXene families, including Ti-, V-, Nb-, and Zr-based compositions, in both pristine and composite forms. We address their application in different types of supercapacitors, such as electric double-layer capacitors (EDLCs), pseudocapacitors, and hybrid devices. The selection of studies was based on their scientific relevance, diversity of electrochemical systems, and methodological rigor, with emphasis on works that explore structure–property relationships, surface chemistry, and performance metrics using experimental and computational approaches.

## 2 Synthesis and Preparation of MXene

Understanding MXene synthesis and their structure–property relationships is key to high-performance electrodes. The predominant route is chemical etching of the A element from MAX phases.^16,21,22^ When the M–A bonds are weaker than the M-X bonds, the selective removal of the A element from the MAX phase is facilitated. During this process, the M–A bonds are broken, while the M-X bonds remain intact, preserving the layered structure characteristic of MXene. This behavior is essential for MXene formation, as it allows the removal of the A element without compromising the stability of the crystalline lattice formed by the M–X bonds.^16,21,22^ This process can be achieved through various chemical approaches, each with its own advantages and limitations, as will be discussed below.

### 2.1 HF-Based Synthesis Method

In the solution-based method, reactants are dissolved in a solvent to promote reaction.^23,24^ For MXene, this method commonly involves the use of hydrofluoric acid (HF). This approach is widely adopted because it simultaneously enables the functionalization of MXene surfaces with groups like –O, –OH, or –F.^12^ Such functionalization is critical for tailoring the properties of MXene, including their hydrophilicity and interactions with other materials, and is necessary due to the strength of the metallic bonds present in the MAX phase.^25^

The methodology employed by Naguib et al.^12^ for obtaining Ti_3_C_2_, one of the first discovered MXene, exemplifies this process. It can be described by the following chemical equations

$$Ti_{3}AlC_{2}+3HF\to AlF_{3}+Ti_{3}C_{2}+\frac{3}{2}H_{2}$$

$$Ti_{3}C_{2}+H_{2}O\to Ti_{3}C_{2}{\left(OH\right)}_{2}+H_{2}$$

$$Ti_{3}C_{2}+2HF\to Ti_{3}C_{2}F_{2}+H_{2}$$

In the first stage, HF removes Al from the MAX phase, forming aluminum fluoride (AlF_3_) and releasing Ti_3_C_2_. Subsequent reactions with water or HF functionalize the MXene surface with –OH and –F groups, respectively, optimizing its structure for specific applications. In Figure 2 it is possible to observe the MXene nanosheets synthesized by Naguib and his collaborators.^12^

**Figure 2:**
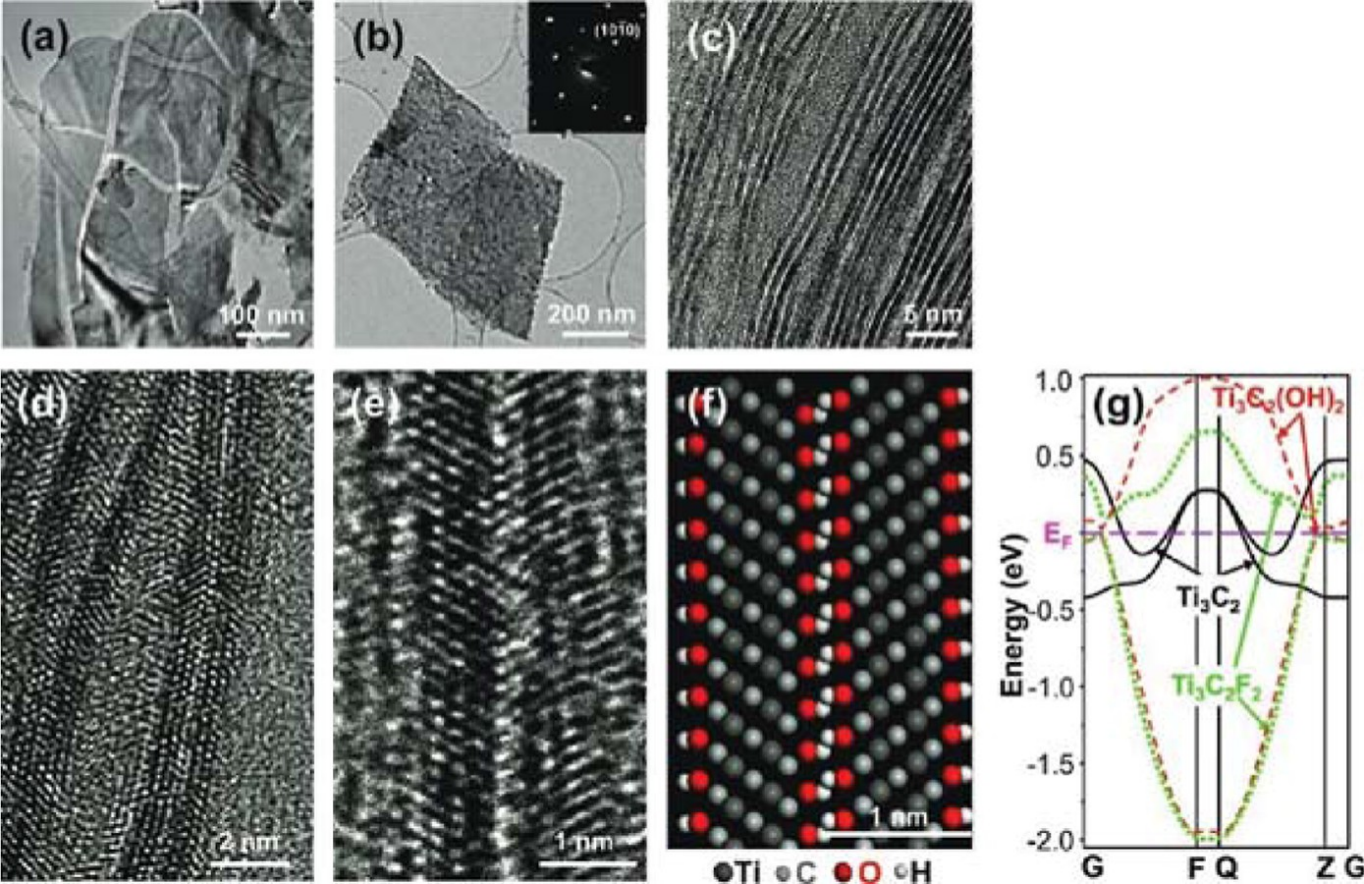
This figure presents the structural and morphological characterization of exfoliated MXene nanosheets. Panel (a) shows a TEM image of 2D nanosheets composed of Ti–C–O–F, while panel (b) displays additional exfoliated sheets; the inset reveals the selected area electron diffraction (SAED) pattern that confirms the hexagonal symmetry of the crystalline planes. In panel (c), single- and double-layer MXene sheets are observed, and panel (d) presents an HRTEM image demonstrating the separation of individual sheets after the sonication process. Panel (e) shows a bilayer Ti_3_C_2_(OH)_*x*_F_*y*_ structure through HRTEM, with panel (f) illustrating the corresponding atomistic model. Finally, panel (g) depicts the calculated electronic band structure of single-layer Ti_3_C_2_ with −OH and −F surface terminations, as well as the pristine form, highlighting the transition from metallic to semiconducting behavior due to surface chemistry modifications. Reproduced with permission from Naguib et al.^12^ Copyright © 2013 John Wiley & Sons, Inc.

Using the same method, Lin et al.^26^ synthesized the MXene Ta_4_C_3_ from the MAX phase Ta_4_AlC_3_, considering the following chemical reactions

$$Ta_{3}AlC_{2}+3HF\to AlF_{3}+Ta_{4}C_{3}+\frac{3}{2}H_{2}$$

$$Ta_{4}C_{3}+2H_{2}O\to Ta_{4}C_{3}{\left(OH\right)}_{2}+H_{2}$$

$$Ta_{4}C_{3}+2HF\to Ta_{4}C_{3}F_{2}+H_{2}$$

$$Ta_{4}C_{3}+O_{2}\to Ta_{4}C_{3}O_{2}$$

However, it is crucial to note that the effectiveness of this process strongly depends on the M–A bond energy, as well as other factors such as HF concentration, etching time, and reaction temperature.^27^ For instance, in the case of the MAX phase Ti_3_AlC_2_, the process can be efficiently carried out using a low HF concentration (5%) over 24 h.^28^ In contrast, for other transition metals like V or Nb, the required HF concentration can be much higher, around 50% wt.^29,30^ While increasing the HF concentration can reduce etching time, it may also cause significant damage to the lateral structure of the synthesized MXene, compromising its integrity.^27,28^ Therefore, precise control of these parameters is essential to ensure the quality and efficiency of the synthesis process.

### 2.2 Alkali-Based Method Synthesis

To mitigate the environmental risks associated with the excessive use of fluorides, the scientific community has been actively developing and studying alternative synthesis methods for MXene. One such method, which aims to both reduce fluoride-related harm and enhance the ability to produce materials with tunable properties, is the alkali-based synthesis method.^31–34^ This approach employs alkaline compounds, such as hydroxides or alkali metal salts (e.g., NaOH, KOH, or LiCl), as substitutes for conventional fluoride agents.^31–34^ It provides a more sustainable pathway for synthesizing MXene while maintaining the efficiency of the process and allowing for greater control over the properties of the resulting materials.^21,31–34^

As an example, we can highlight the work by Kulkarni et al.,^31^ where the authors synthesized MXene using a safer and more efficient synthesis method based on an alkaline environment with potassium hydroxide (KOH). The MAX precursor Ti_3_AlC_2_ was subjected to a hydrothermal etching process, where it was treated with KOH solutions under controlled conditions. Various KOH concentrations, reaction times, and precursor pretreatments were explored to optimize the quality of the produced MXene. The study revealed that the KOH concentration and reaction time significantly influence the etching efficiency and MXene stability. However, pretreatments at high temperatures and prolonged reaction times can lead to the formation of titania nanofibers, degrading the material’s quality. The sample synthesized with 15 M KOH for 24 h at 180 °C showed the best performance in terms of quality and electrochemical properties. MXene characterization was performed using advanced techniques such as X-ray photoelectron spectroscopy (XPS) and Fourier-transform infrared spectroscopy (FTIR), which confirmed the presence of functional groups on the material’s surface, such as −OH and Ti–O, indicating successful MXene functionalization. Furthermore, the authors fabricated MXene composites with poly­(3,4-ethylenedioxythiophene):polystyrenesulfonate (PEDOT:PSS), which served as a conductive medium, enhancing the material’s dispersion and stability. These composites demonstrated significant potential as active materials for supercapacitor electrodes, achieving specific capacitances exceeding 1000 F/cm^3^ and excellent capacity retention after 10,000 cycles.

In the study conducted by Guo et al.,^32^ the chemical etching behavior of Mo_2_Ga_2_C was analyzed using three different alkaline solutions: LiOH, NaOH, and KOH, at three different concentrations (10, 15, and 20 M). The researchers demonstrated that the MXene Mo_2_C can be successfully synthesized through the alkaline-based synthesis method, using a 20 M NaOH solution at 180 °C for 24 h. This study was the first to achieve MXene synthesis using this approach. The findings revealed that, compared to the standard chemical etching process with HF, the Mo_2_C MXene synthesized in NaOH solution exhibited significant advantages. The material showed a larger specific surface area due to the intercalation of Na^+^ ions, as well as lower electrical resistance, attributed to the presence of Na^+^ residues and pure surface terminations of the –O and –OH types, without any fluoride contamination. Furthermore, the authors evaluated the performance of the Mo_2_C MXene as an anode for lithium-ion batteries (LIBs). The MXene produced with NaOH achieved a final specific capacity of 266.73 mA h·g^–1^ at a current density of 0.8 A·g^–1^, representing a 52% increase compared to the MXene synthesized with HF. These results highlight the potential of alkaline-based methods for synthesizing MXene with superior electrochemical properties.

Li et al.^33^ demonstrated the synthesis of the MXene Ti_3_C_2_(OH)_2_ through the treatment of Ti_3_AlC_2_ with KOH in a system containing a small amount of water. During this process, it was observed that the Al layer within the original structure was removed and replaced by –OH groups. Additionally, the synthesized material exhibited great potential for applications in energy storage devices. In contrast, ref 34 reported the successful preparation of Ti_3_C_2_T_*x*_ using a treatment with 27.5 M NaOH at 270 °C. The researchers highlighted that the alkali-based synthesis method did not result in surface terminations containing fluorine. The produced material stood out for its high gravimetric capacitance of 314 F/g and volumetric capacitance of 511 F/cm^3^ at a scan rate of 2 mV/s. When compared to Ti_3_C_2_T_*x*_ produced by HF-based methods, the NaOH-synthesized material demonstrated a performance improvement of 214%. This result underscores the significant potential of alkali-based synthesis methods for producing MXene with optimized properties for energy storage applications. Figure 3 shows the characterization of MXene Ti_3_C_2_T_*x*_ obtained using the NaOH synthesis method, its analysis spectra and the characterizations of its microstructures.^34^

**Figure 3:**
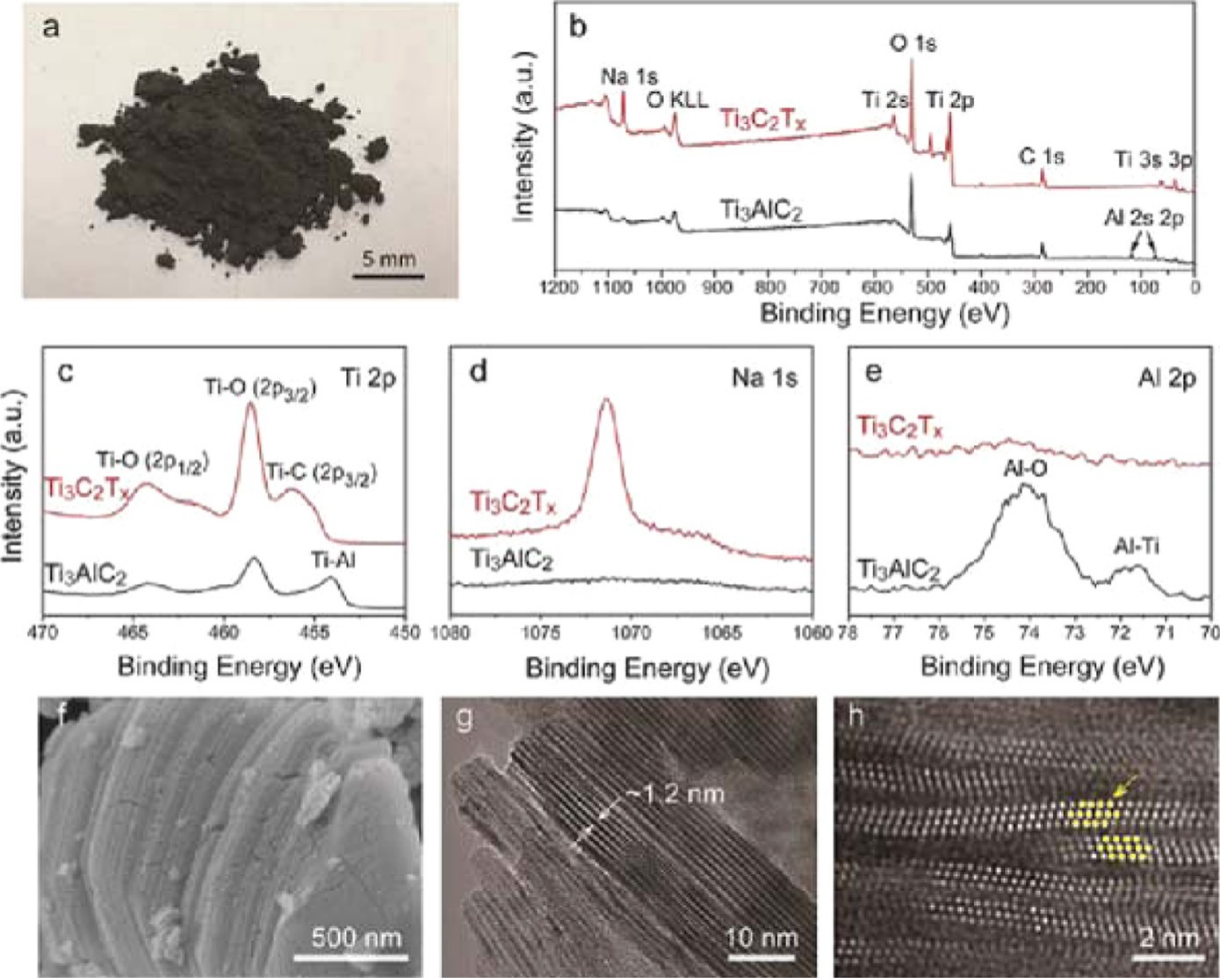
Characterization of the Ti_3_C_2_T_*x*_ obtained using 27.5 M NaOH under 270 °C. Panel (a) shows the resulting Ti_3_C_2_T_*x*_ powder. Panels (b–e) display the X-ray photoelectron spectroscopy (XPS) spectra comparing the pristine Ti_3_AlC_2_ and the synthesized Ti_3_C_2_T_*x*_ including (b) the full survey, (c) The Ti 2p region, (d) the Na 1s region, and (e) the Al 2p region, respectively. Panels (f) to (h) provide microstructural characterizations of the Ti_3_C_2_T_*x*_ flakes using (f) scanning electron microscopy (SEM), (g) transmission electron microscopy (TEM), and (h) high-angle annular dark-field scanning transmission electron microscopy (HAADF-STEM), respectively. Where the bright spots in panel (h) correspond to titanium atom positions. Reproduced with permission from Li et al.^34^ Copyright © 2018 John Wiley & Sons, Inc.

### 2.3 Electrochemical Etching Synthesis Method

In addition to the methods discussed in the previous subsections, the electrochemical etching synthesis method stands out. This method involves applying a constant potential difference in an acidic medium, typically containing Cl^–^ ions, which exhibit a high affinity for forming stable combinations with elements like Al.^21,35,36^ During the process, a controlled voltage is applied to selectively remove the A atoms (usually Al) from the MAX precursor. This removal occurs due to the preferential oxidation of Al at the anode, facilitating the extraction of the A element without the need for highly corrosive chemical agents such as HF, which is commonly used in traditional methods.^21^ This approach not only reduces the environmental and operational risks associated with HF but also allows for greater precision in the synthesis process. The chemical reaction for the MAX precursor can be expressed as

$$Ti_{3}AlC_{2}\to Ti_{3}C_{2}T_{x}+Al^{3+}$$

where Al is oxidized to Al^3+^ ions, while the Ti_3_C_2_ structure is preserved. This methodology represents a promising and more sustainable alternative for MXene production, with potential to improve both safety and efficiency.

This method was applied by Yin et al.,^35^ who synthesized the Ti_3_C_2_F_*x*_ material using an electrochemical exfoliation and delamination process, which proved to be more effective than traditional methods involving hydrofluoric acid (HF). The synthesis was conducted in an electrochemical system comprising a working electrode made of TiAlC, a platinum (Pt) counter electrode, and a silver (Ag) reference electrode. The electrolyte used was a nonaqueous system composed of [BMIM]­[PF_6_] (1-butyl-3-methylimidazolium hexafluorophosphate) and MeCN (acetonitrile), which was carefully purified prior to use to ensure reaction stability and purity. During the process, a constant potential ranging from +3 to +7 V was applied to facilitate the reaction between fluoride anions in the electrolyte and aluminum atoms in the MAX phase, leading to material exfoliation. The study identified +5 V as the optimal potential to prevent overexfoliation of the MAX precursor, ensuring a well-defined and functional structure. Higher potentials, such as +7 V, resulted in the formation of amorphous species and carbon deposition on the surface, compromising the quality of the synthesized material. This research underscores the effectiveness of the electrochemical method for producing MXene with controlled properties, while avoiding the hazards associated with HF use.

In the study conducted by Liu et al.,^36^ a pellet containing stoichiometric amounts of a MAX precursor composed of Ti, C, and Al was prepared using different carbon sources: graphite, carbon nanotubes (CNT), and reduced graphene oxide (rGO). The pellet was coated with Mo to ensure electrical contact and placed in a molten salt cell. The treatment was performed at specific temperatures depending on the carbon source used (1000 °C for graphite, 970 °C for CNT, and 950 °C for rGO) for a determined duration. After thermal treatment, the cell was cooled and connected to a potentiometer for the electrolysis step. During this phase, potentials of 0.8 V for rGO and 1.3 V for CNT were applied to selectively oxidize the Al atoms in the MAX precursor, resulting in the formation of the MXene Ti_2_CT_*x*_. The materials were characterized using X-ray diffraction (XRD), showing similar patterns for MXene derived from rGO and CNT, indicating high crystallinity as can see in the Figure 4.^36^ However, minor phase variations were observed, suggesting the formation of Ti_3_C_2_Cl_*x*_ as a byproduct. Despite this, the synthesized MXene exhibited excellent charge and discharge capacities, underscoring their significant potential for energy storage device applications.

**Figure 4:**
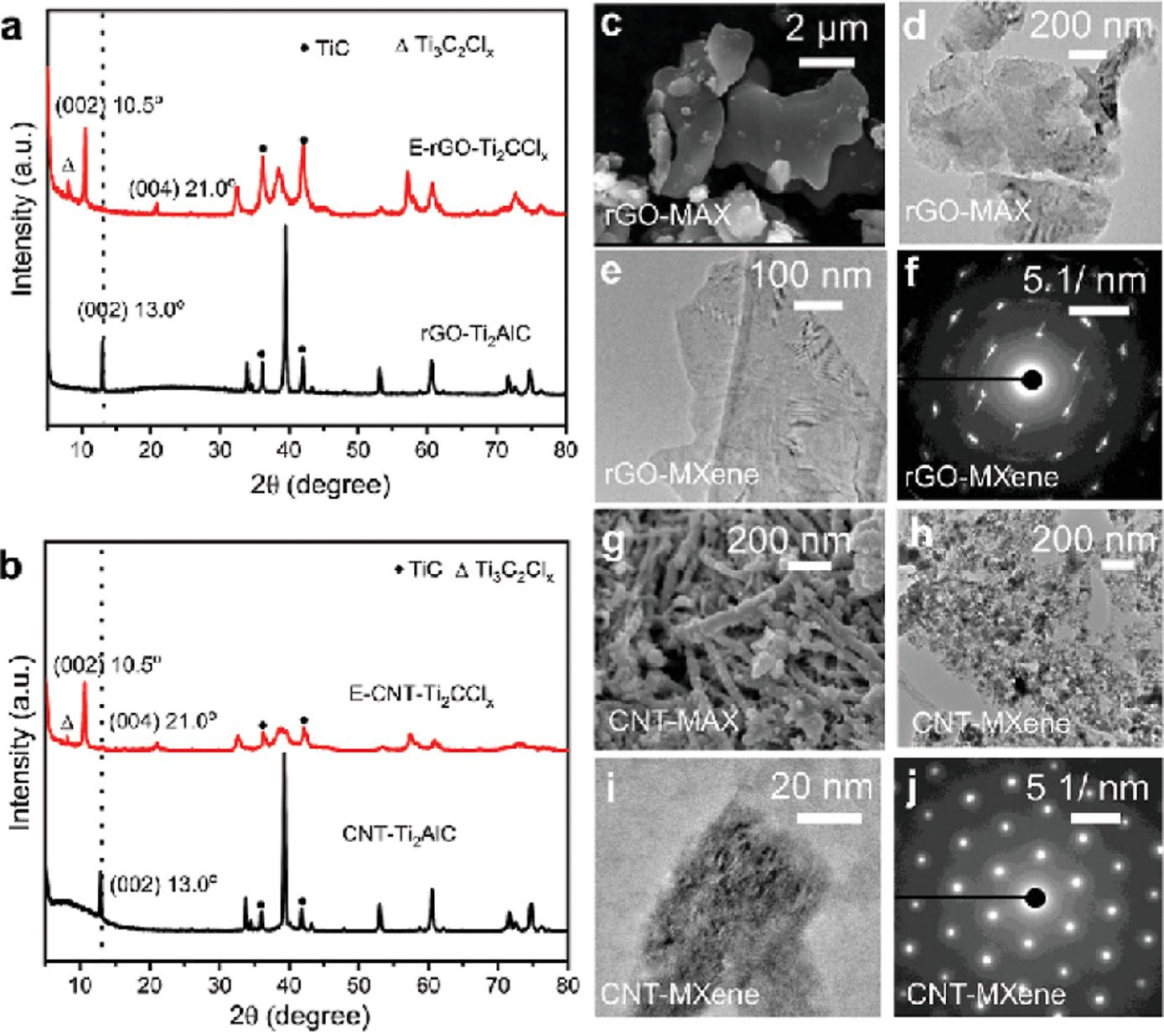
Structural characterizations of Ti_2_AlC MAX phase and the corresponding E-Ti_2_CCl_*x*_ MXene synthesized using two types of carbon sources: two-dimensional reduced graphene oxide (rGO) and one-dimensional carbon nanotubes (CNT) carbon sources. Panels (a,b) show the X-ray diffraction (XRD) patterns for MAX and MXene derived from rGO and CNT, respectively. For the rGO-Ti_2_AlC MAX phase, panels (c,d) display SEM and TEM images, while panels (e,f) show the TEM image and selected area electron diffraction (SAED) pattern of the resulting E-rGO-Ti_2_CCl_*x*_ MXene. Panels (g–j) correspond to the CNT-based system: (g) shows SEM images of the CNT-Ti_2_AlC MAX phase at a scale of 200 and 20 nm, respectively; and (j) presents its corresponding SAED pattern. Reproduced with permission from Liu et al.^36^ © 2023 John Wiley & Sons, Inc.

### 2.4 Molten Salt Etching Method

The molten salt synthesis method is widely recognized for preparing advanced materials, including carbon, perovskites, metal oxides, fluorides, and other inorganic solids.^37–39^ Generally, this method employs a eutectic mixture of salts, such as LiCl/KCl or NaCl/KCl, which act as a reactive medium.^38^ Given the increasing interest in developing more efficient MXene synthesis methods, molten salt synthesis shows significant potential for application.

In MXene preparation, the molten salt is mixed with the MAX phase precursor at high temperatures (typically near the melting point of the salts). During this process, the salt facilitates the removal of the A element from the MAX phase, resulting in the formation of MXene. For instance, in the study by Li et al.,^40^ several MAX phases (Ti_3_ZnC_2_, Ti_2_ZnC, Ti_3_ZnN, and V_2_ZnC) and MXene Ti_3_C_2_Cl_2_ and Ti_2_CCl_2_ were successfully synthesized by substituting the A element in the MAX phase using molten ZnCl_2_.

In another study by Shen et al.,^41^ the authors combined molten salt and electrochemical etching methods to synthesize Ti_3_C_2_Cl_2_ MXene. In their approach, the MAX phase precursor served as the anode, and nickel as the cathode in an electrochemical cell containing a LiCl–KCl molten salt mixture at 450 °C as the electrolyte. When a 2 V potential difference was applied in the presence of Cl^–^ ions, Al atoms were converted to AlCl_3_, making them easier to oxidize under the high-temperature, high-voltage conditions. Consequently, the oxidized Al atoms evaporated, resulting in the formation of Ti_3_C_2_Cl MXene. The authors further noted that at higher voltages (above 3 V), TiCl_4_ formation occurred, whereas at lower voltages (below 1.6 V), the MAX phase remained unchanged. Figure 5 summarizes the MXene synthesis process using this method.

**Figure 5:**
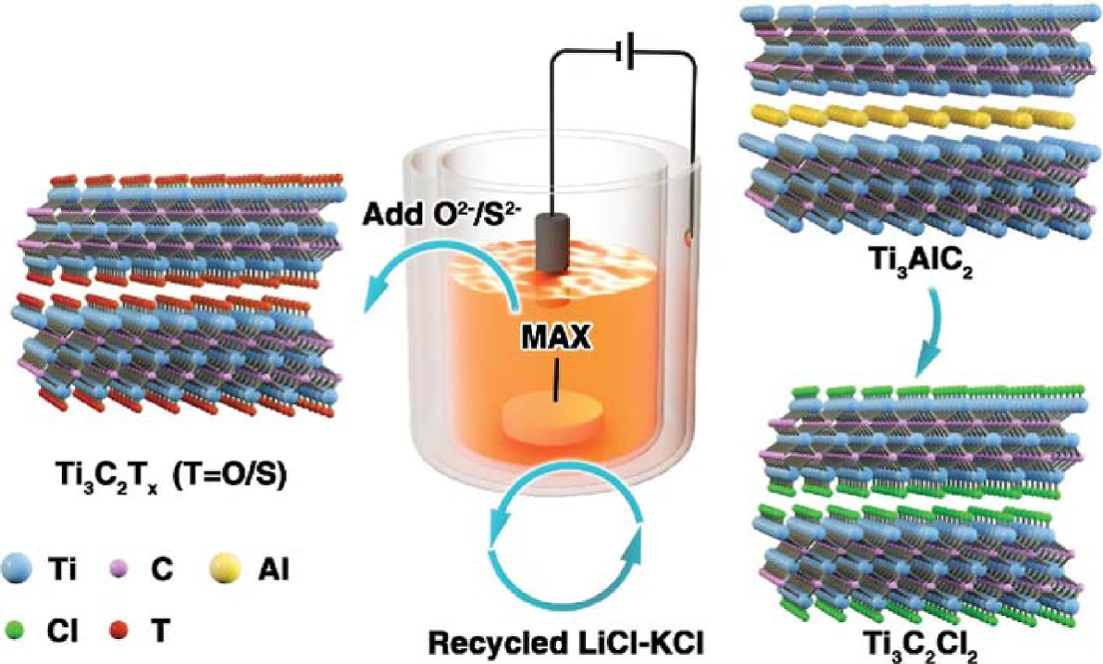
Schematic illustration of the MXene synthesis process from the MAX phase using the molten salt electrochemical etching (MS-E-etching) technique, along with the in situ modification of surface terminations. This method enables controlled etching and functionalization of the MXene structure during synthesis. Reproduced with permission from Shen et al.^41^ Copyright © 2021 John Wiley & Sons, Inc.

### 2.5 sHydrothermal Synthesis Method

To mitigate the environmental impacts associated with conventional synthesis methods that use HF, the hydrothermal synthesis method emerges as an excellent alternative for MXene production. Peng et al.^42^ developed an innovative approach to synthesize Ti_3_C_2_ and Nb_2_C using a mixture of sodium tetrafluoroborate (NaBF_4_) and hydrochloric acid (HCl). In their study, 0.75 g of NaBF_4_ was dissolved in 15 mL of HCl (37 wt %), and 0.25 g of the MAX phase Ti_3_AlC_2_ was added to the solution and mixed uniformly. The mixture was then transferred to an autoclave and treated at 180 °C for 8 to 32 h. Similarly, for Nb_2_C MXene, 0.5 g of the MAX phase Nb_2_AlC was uniformly mixed in 15 mL of HCl (37 wt %) containing 0.75 g of NaBF_4_, and the hydrothermal reaction was carried out at 180 °C for 15 to 35 h. After the reactions, the products were washed with deionized water and dried at 70 °C under vacuum, yielding Ti_3_C_2_ and Nb_2_C MXene. Figures 6 and 7 depict the products of the hydrothermal reactions, corresponding to the Ti_3_C_2_ and Nb_2_C MXene obtained through the described procedures.

**Figure 6:**
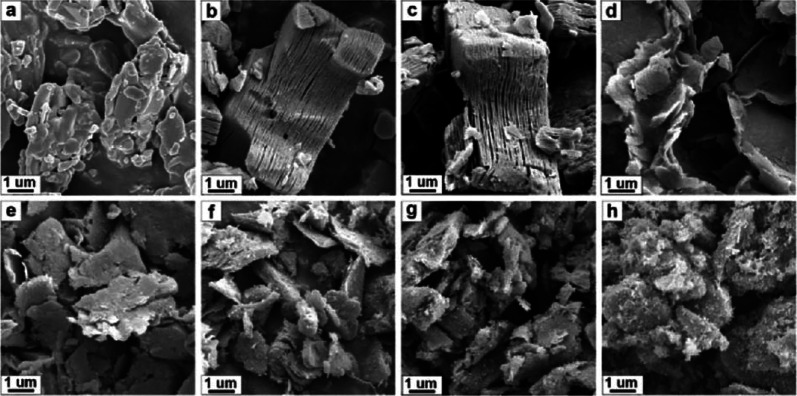
Scanning electron microscopy (SEM) images showing the morphology of the Ti_3_AlC_2_ MAX phase (a) and evolution of the h-Ti_3_C_2_ MXene structure as a function of hydrothermal reaction time: (b) 8 h, (c) 12 h, (d) 16 h, (e) 20 h, (f) 24 h, (g) 28 h, and (h) 32 h. Reproduced with permission from Peng et al.^42^ Copyright © 2018 Elsevier. Originally published as Figure 2.

**Figure 7:**
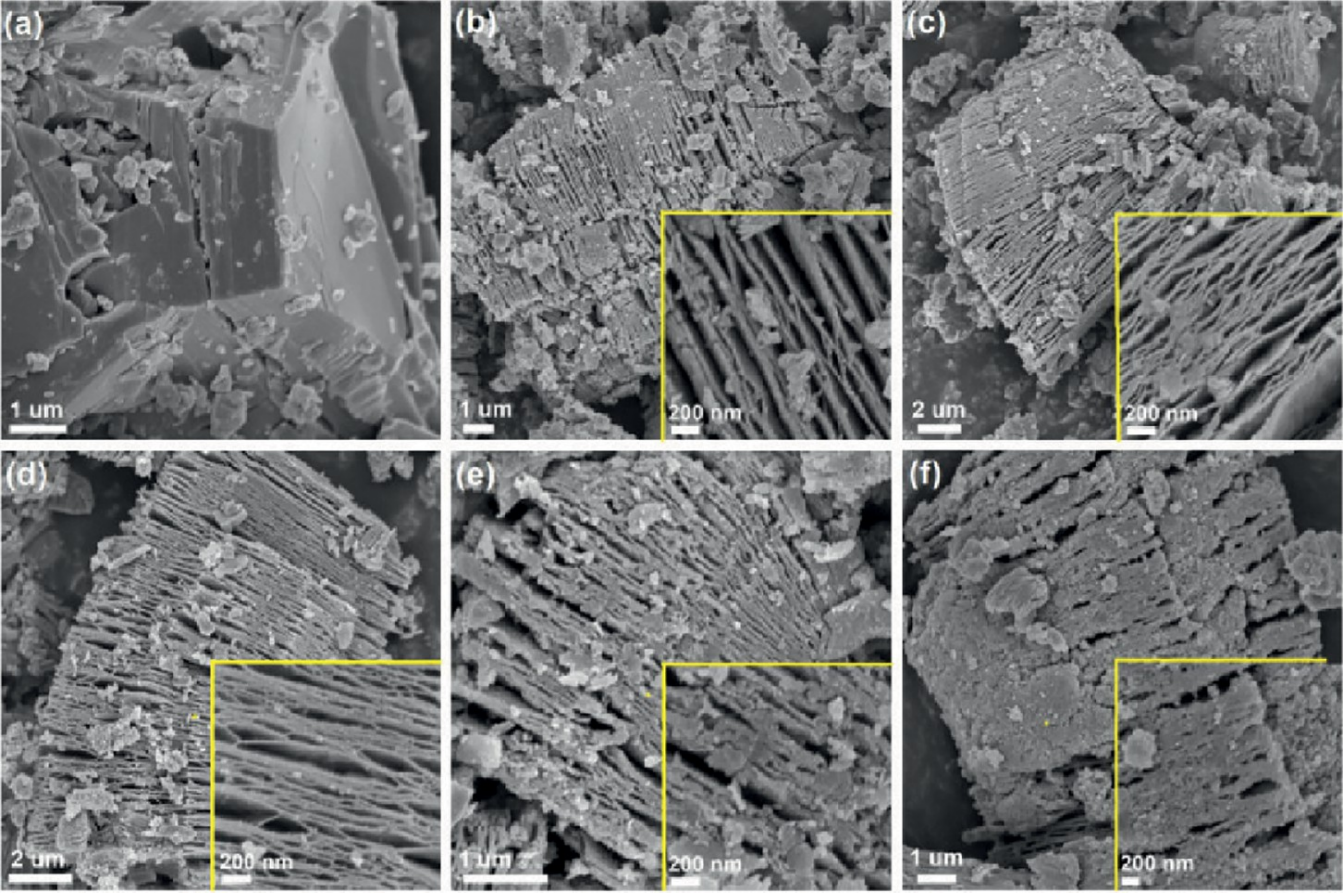
SEM images of Nb_2_AlC MAX phase (a) and hydrothermally treated h-Nb_2_C MXene synthesized using varying reaction times: (b) 15 h, (c) 20 h, (d) 25 h, (e) 30 h and (f) 35 h. Reproduced with permission from Peng et al.^42^ Copyright © 2018 Elsevier. Originally published as Figure 8.

Furthermore, the authors compared the hydrothermal synthesis method with the traditional HF-based method. The results demonstrate that the hydrothermal synthesis method can be more efficient than the conventional approach. For Ti_3_C_2_ MXene produced via the hydrothermal process, a larger lattice parameter and greater interlayer spacing were observed compared to MXene synthesized using the conventional method. This behavior was also observed for Nb_2_C MXene synthesized via the hydrothermal process, highlighting the higher efficiency of the hydrothermal route in exfoliating Al atoms from the MAX phase.

Other studies^43,44^ have also reported success in synthesizing MXene through the hydrothermal method. Wang et al.^43^ synthesized Ti_3_C_2_T_*x*_ MXene by exfoliating the MAX phase using NH_4_F as a reagent. The authors dissolved 5 g of NH_4_F in 60 mL of water to create a homogeneous solution and then added 0.5 g of the MAX phase under rapid stirring. The resulting mixture was transferred to an autoclave and treated at 150 °C for 24 h. Under these high-temperature and high-pressure conditions, exfoliation of the MAX phase occurred, resulting in the formation of MXene. In another study, Guo et al.^44^ used the MAX precursor Mo_2_GaC to produce Mo_2_CT_*x*_ MXene through the hydrothermal method. The authors employed different chemical mixtures to optimize the process, demonstrating the versatility of the hydrothermal method for synthesizing MXene based on various transition metals.

## 3 Results of MXene-Based Materials in SCs

In this section, we will present the main results from the literature regarding the application of MXene in supercapacitors, encompassing both computational and experimental studies. These works provide a comprehensive overview of the electrochemical performance, energy storage mechanisms, and optimization strategies to enhance the efficiency of these materials in energy storage devices.

### 3.1 Computational Results

Computational simulation studies play a crucial role in the development of new technologies and, most importantly, new materials. These simulations enable the assessment of a material’s feasibility for applications in human-relevant scenarios, such as energy storage. Through computational methods, it is possible to investigate fundamental properties, predict behavior under various conditions, and guide experiments to optimize material performance, thereby accelerating scientific and technological progress.

This role has been explored in various studies, such as the one conducted by Sun et al.,^45^ where two MXene with different surface terminations (Ti_3_C_2_O_2_ and Ti_3_C_2_(OH)_2_) were investigated as electrodes in combination with the ionic liquid (IL) [HEMIm]­[NTf_2_] in a theoretical SC model. Using molecular dynamics (MD) simulations, the authors varied the interlayer spacing of the electrodes and calculated the device’s electrical properties. The OPLS-AA force field was employed, and electrode molecule parameters were derived from prior studies.^46,47^ The results showed that an interlayer spacing of 1.4 nm led to a nearly 25% increase in capacitance compared to a spacing of 1 nm under an applied voltage of 2 V. Interlayer spacing variation directly influenced the structure of the electric double layer (EDL), affecting ion density and charge distribution. Regarding surface terminations, −OH groups on the MXene had a significant impact on ion orientation in the EDL. Near the electrode surface, cations tended to align vertically, facilitating the retention of more cations. This behavior was attributed to the formation of hydrogen bonds (O–H···O) between the −OH groups of the MXene and the −OH groups of the ILs, contributing to the preferential orientation of the ions. Differential capacitance was calculated based on surface electron density. The results indicated that the Ti_3_C_2_(OH)_2_ electrode exhibited superior differential capacitance compared to Ti_3_C_2_O_2_, suggesting that stronger hydrogen bonding supports the vertical aggregation of cations, optimizing charge storage. Furthermore, the analysis of ion and charge density in confined spaces revealed the formation of a robust EDL at the interface between the MXene electrode and the IL electrolyte. Both interlayer spacing and surface terminations played crucial roles in the EDL structure, emphasizing the importance of optimizing these features to enhance supercapacitor performance. These findings provide fundamental insights into the mechanisms governing ion–electrode interactions in MXene, suggesting that manipulating interlayer spacing and surface terminations can be an effective strategy for developing high-performance supercapacitors. The study highlights the need for further theoretical investigations to thoroughly explore the effects of structural and interfacial characteristics on energy storage devices.

In the study conducted by Xu et al.,^48^ molecular dynamics simulations were performed to investigate a system consisting of Ti_3_C_2_(OH)_2_ electrodes and the conventional ionic liquid [EMIM]­[PF_6_], with varying electrode distances. The primary goal was to determine the number of ions inserted into the electrode nanopores in the neutral state. It was found that electrolyte ions could spontaneously insert into the electrode nanopores at all distances analyzed. The polar surface of the Ti_3_C_2_(OH)_2_ MXene rendered the nanopores ionophilic, promoting efficient use of the internal pore surface. Moreover, the total number of ions in the region increased with the nanopore volume. An intriguing phenomenon was observed when analyzing the system’s net charge: electroneutrality in the nanopores was disrupted due to the preferential adsorption of [EMIM]^+^ over [PF_6_]^−^. This resulted in a positive net charge (4.4 e^–^, 2.8 e^–^, and 1.8 e^–^ for pores of 0.7, 1.0, and 1.4 nm, respectively). This asymmetry was attributed to stronger specific interactions between cations and the electrode. In aqueous carbon-based systems, the situation can reverse, with anions being more strongly adsorbed, leading to a negative net charge, as also observed in experimental studies.^49,50^ The diffusion coefficient of [EMIM] and [PF_6_] ions varied throughout the process, showing an initial increase during charging, followed by a decrease as the pores became more congested. Ion diffusion was more efficient in smaller pores (0.7 nm) compared to larger ones (1.4 nm), with the diffusion coefficient in 0.7 nm pores being approximately twice as high. During discharge periods, diffusion initially increased but then decreased, reflecting the complexity of ionic movement in response to charge changes. Ion density analysis revealed an alternating layered structure of cations and anions within the pore, resulting in local charge separation. This alternating ion distribution contributes to the formation of the electric double layer (EDL), a key feature for electrochemical performance, as it influences charge storage capacity and response to the applied potential. The authors also observed oscillations in the total number of ions in the cathode pore around an equilibrium value, whereas in the anode pore, these oscillations exceeded the equilibrium value. These ion oscillations induced volumetric expansion and contraction between electrode layers, potentially affecting electrode stability and cyclic lifetime. The authors suggested that these volumetric variations during charge and discharge cycles require further investigation, both theoretically and experimentally, to better understand their implications for electrode durability.

Conversely, ref 51 presents a study focused on Ti_2_CT_2_ nanosheets (T = –O, –F, and –OH). The stability of these compounds in aqueous electrolytes was investigated through work function calculations. The authors found Ti_2_CF_2_ sheets stable in aqueous electrolytes, whereas sheets with other terminations are unstable. Additionally, the intrinsic capacitances of Ti_2_CF_2_ and Ti_2_CO_2_ were analyzed through the density of states (DOS), and the impact of electrolyte acidity was also discussed. In sodium-ion hybrid capacitors, the storage capacity, structural changes, and capacitance of Ti_2_CT_2_ materials were studied. The results revealed that redox pseudocapacitance is primarily contributed by Ti_2_C sheets terminated with oxygen. The low energy barriers indicated that sodium ions can move quickly in and out of the material, enabling efficient pseudocapacitance and ensuring good performance even at high charge and discharge rates. The authors employed density functional theory (DFT) calculations using the VASP software.^52^ The ion–electron interaction was described with the Projector Augmented Wave (PAW) method and the PBE exchange–correlation functional within the GGA approximation. The electrochemical capacitance was evaluated by analyzing the increase and decrease in unoccupied states. Additionally, the open-circuit voltage (OCV) was calculated, considering that once the OCV reaches zero, the nanosheets can no longer accommodate Na^+^ ions. The chemical reaction considered was

$$Ti_{3}CT_{2}+2Na^{+}+xe^{-}\to Ti_{2}CT_{2}Na_{x}$$

with the open-circuit voltage (OCV) defined as the energy difference, neglecting volume and entropy effects

$$OCV\approx \left[E\left(Ti_{2}CT_{2}\right)+xE\left(Na\right)\right]-\frac{E\left(Ti_{2}CT_{2}Na_{x}\right)}{xe}$$

The results showed that Ti_2_CO_2_ and Ti_2_CF_2_ nanosheets exhibited capacitances of 291.5 and 252.2 F·g^–1^, respectively, indicating good charge storage performance. The pseudocapacitance observed in Ti_2_CO_2_ nanosheets was attributed to strong interactions with adsorbed cations. It was also found that Ti_2_CF_2_ nanosheets exhibit higher stability in aqueous electrolytes compared to Ti_2_CO_2_ and Ti_2_C­(OH)_2_. Furthermore, the authors highlighted that the electrolyte acidity can affect both the stability and capacitance of the nanosheets. The results suggest that Ti_2_CT_2_ nanosheets hold significant potential for energy storage technologies, offering a promising alternative to conventional materials.

Shifting from electronic to atomistic simulations, Cheng and Sprik^53^ investigated the interface between metal oxide (TiO_2_) and water using density functional theory molecular dynamics (DFTMD) techniques. The study focused on determining the surface p*K*_a_ of TiO_2_ and modeling the electric double layer (EDL) under varying pH conditions. The primary goal was to understand how protonation and deprotonation processes influence the surface charge of TiO_2_ and, consequently, the formation of the EDL. The calculated capacitance was differentiated for inner-sphere and outer–sphere complexes, yielding approximately 0.4 F·m^–2^ and 0.3 F·m^–2^, respectively. These values were considered small compared to experimental data, with a discrepancy of about a factor of 3. This discrepancy was attributed to simplifications in the DFTMD model, such as limitations in the system size, which affected the accuracy of the results. In the same context of atomistic investigations, Xu et al.^54,55^ aimed to understand how volumetric changes in electrodes affect charge storage and the formation of the electric double layer (EDL) in supercapacitors. In ref 54 the investigations were concentrated in SCs composed of Ti_3_C_2_T_*x*_ MXene electrodes and the ionic liquid electrolyte [EMIM]­[TFSI]. Using MD methods, the authors explored the fundamental mechanisms driving the performance of these devices. The results showed that, despite the relatively short simulation times, longer simulations (0.6 ns for charge and discharge processes) do not significantly alter the findings. This was attributed to the small pore size of the electrodes and the high temperature used (450 K), which facilitated rapid ion diffusion in the system. The authors concluded that ionic rearrangements and dynamic volumetric changes in the electrodes are crucial for the performance of supercapacitors. These results highlight how 2D MXene improve storage efficiency and inform future device design. In ref 55 the authors investigated how the nature of functional groups and the size of anions influence charge storage mechanisms and volumetric changes in electrode materials. The study focused on graphene electrodes and Ti_3_C_2_T_2_ MXene (T = –O, –F, and –OH). The investigation employed MD techniques using the OPLS-AA force field to model the interactions between ions and electrodes. Partial charges for MXene atoms were obtained from the literature^56^ to ensure global neutrality of the electrodes in the uncharged state. Additionally, five ionic liquids were studied, each with a common cation (1-ethyl-3-methylimidazolium, EMIM^+^) and anions of varying sizes: Cl^–^, BF_4_^–^, OTF^–^, FSI^–^, and TFSI^–^. The findings revealed distinct charge storage mechanisms between graphene electrodes and MXene. MXene exhibited a stronger dependence on the characteristics of surface functional groups and ion size compared to graphene. Charge storage efficiency in MXene was significantly influenced by surface charges, and the number of ions in the interlayer region varied substantially during charge and discharge processes. In graphene electrodes, the number of cations remained consistent, whereas, in MXene, both anions and cations exhibited significant variation depending on ion size. These results suggest that MXene offer advantages in terms of customization and optimization for energy storage in SCs. The greater influence of functional group characteristics and ion size on MXene highlights their potential for adaptable performance in various applications.

In addition to these studies, a noteworthy investigation was conducted by Sampaio et al.,^57^ who studied SCs with Ti_3_C_2_F_2_-based electrodes using two different ILs as electrolytes: [EMIM]­[NTf_2_] and [EMIM]­[BF_4_]. The study analyzed two distinct electrode configurations: one with electrodes positioned at the edges and another with electrodes located within the liquid itself as can be seen in Figure 8.

**Figure 8:**
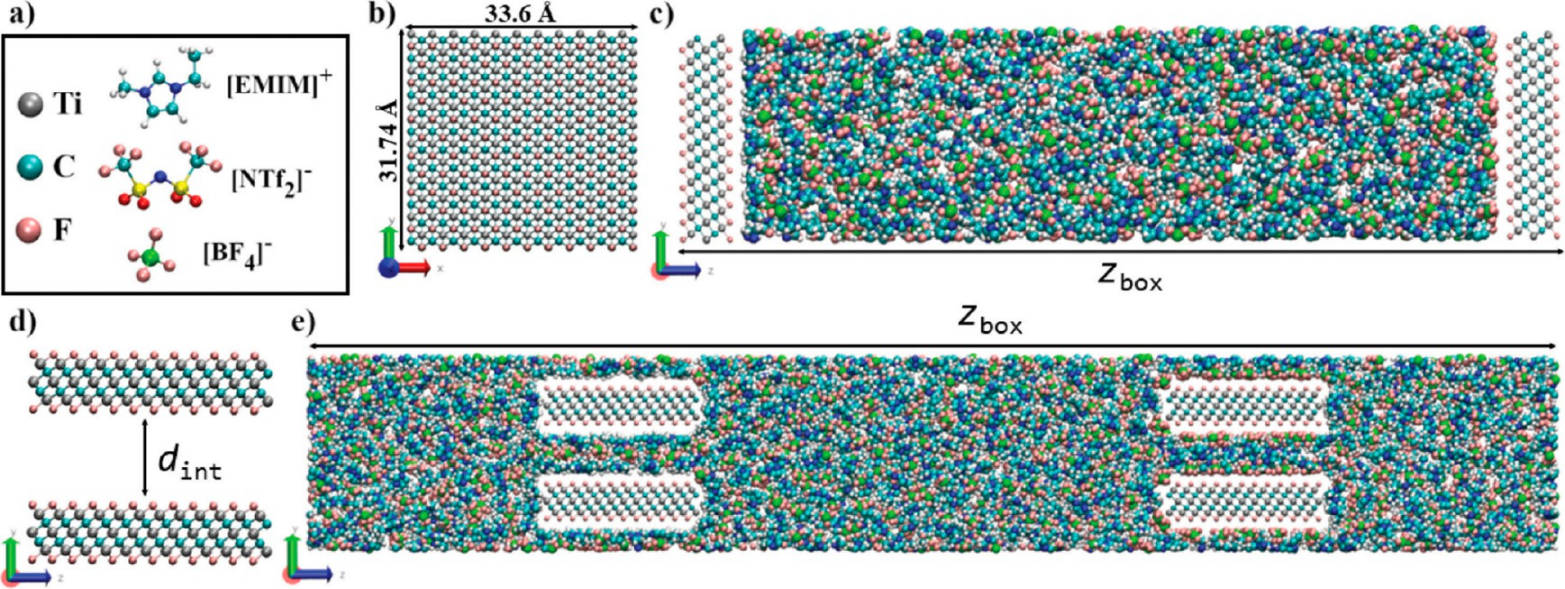
Schematic representation of key components and configurations involved in Ti_2_C_2_F_2_-based SCs. Panel (a) depicts the chemical structures of the ionic liquids [EMIM]­[NTf_2_] and [EMIM]­[BF_4_]. Panel (b) shows the dimensions of an individual Ti_3_C_2_F_2_ layer, while panel (c) presents a planar SC configuration using Ti_3_C_2_F_2_ electrodes. Panel (d) illustrates the interlayer spacing between two Ti_3_C_2_F_2_ layers; and panel (e) displays the SC constructed with layered Ti_3_C_2_F_2_ electrodes. Reproduced with permission from Sampaio et al.^57^ Copyright © 2024 Elsevier. Originally published as Figure 1.

The primary methodology involved MD simulations using the constant potential method (CPM), applying a potential difference of 3 V. The authors demonstrated that the constant potential method is highly effective for evaluating the electrical properties of supercapacitors, yielding results consistent with existing literature, mainly to analyze how the charge behaves along the electrodes. The specific capacitance values were calculated considering charges on all electrode atoms. For the [EMIM]­[NTf_2_] electrolyte, the capacitance ranged from 26.5 F/g to 32.6 F/g, while for the [EMIM]­[BF_4_] electrolyte, values ranged from 30.2 F/g to 40.6 F/g. These findings highlight the significant impact of the electrolyte type on supercapacitor efficiency and underscore the robustness of the CPM method as a powerful tool for analyzing energy storage devices.

In ref 58 Sun and collaborators investigated the two-step charging process in sulfuric acid (H_2_SO_4_) electrolytes using molecular dynamics simulations to understand the energy storage mechanisms in electrodes composed of MXene and MXene/graphene. The focus was to analyze how the combination of these materials could enhance supercapacitor performance. A charge and discharge process were simulated with surface charge densities ranging from 0 to 60 μC/cm^2^ and 60–0 μC/cm^2^. During the charging process, the energy of the anode made of pure MXene decreased with increasing charge density, from an initial value of −73.40 kJ/mol to a final value of −648.35 kJ/mol, representing a variation of 574.95 kJ/mol. Similarly, the anode composed of MXene/graphene showed the same trend, with initial and final values of −139.09 kJ/mol and −734.57 kJ/mol, respectively, resulting in a variation of 595.48 kJ/mol. For the cathode made of pure MXene, a relatively stable behavior was observed, with an energy variation of 154.15 kJ/mol. However, the behavior of the cathode composed of MXene/graphene was more complex, with the energy decreasing rapidly at low charge densities, followed by fluctuations of increases and decreases, resulting in a total variation of 474.25 kJ/mol. The study provided valuable insights into energy storage, showing that the performance of supercapacitors using composite electrodes can be significantly better compared to those with pure MXene electrodes, highlighting their potential for advanced energy storage applications.

It is notable that studies investigating MXene as electrodes in SCs using MD techniques often employ the CPM. This method is particularly suitable for metallic materials, such as MXene, as their metallic properties significantly affect ionic movements near the electrode surfaces. CPM allows the observation of charge fluctuations across electrodes, ensuring that all atoms remain at the same potential, as expected for conductive materials.^59^ However, CPM has limitations, such as the lack of incorporation of atomic electronegativity.^60^ To address this, Lin et al.^61^ proposed a novel heteroatomic constant potential method (HCPM) for studying energy storage systems with MXene-based and/or other heteroatomic conductive electrodes. The authors conducted simulations of Ti_3_C_2_(OH)_2_ MXene with a Li-TFSI/AN electrolyte using the OPLS-AA force field. The findings demonstrated that the HCPM is highly suitable for systems involving heteroatomic electrodes, as it incorporates electronegativity effects and charge response adjustments, leading to more realistic simulation results. Moreover, the method adds minimal computational cost compared to conventional methods. However, the authors noted that HCPM still has limitations, such as the need for improved interaction parameters, which could be developed through more detailed calculations, such as those based on DFT.

There is a strong preference for titanium-based MXene, particularly Ti_3_C_2_T_*x*_, due to several factors: (1) ease and cost of preparation: the synthesis process of Ti_3_C_2_T_*x*_ is relatively simple and cost-effective, offering excellent potential for commercial scalability.^62,63^ (2) Structural flexibility: Ti_3_C_2_T_*x*_ is highly versatile in terms of structures, being producible as three-dimensional multilayers, two-dimensional monolayers, and even zero-dimensional quantum dots, greatly expanding its range of applications.^63,64^ (3) Tunable physicochemical properties: the electronic band structure and physicochemical properties of Ti_3_C_2_T_*x*_ can be easily controlled by modifying its surface functional groups.^63,65^ (4) Local defects: In addition to surface terminations, Ti_3_C_2_T_*x*_ contains local defects that can be fully utilized for the preparation of advanced MXene-based composites.^63,66^ Ti_3_C_2_T_*x*_ also exhibits extraordinary electrical conductivity even with surface terminations and structural defects^63,67^ and, mainly (5) Biocompatibility and biodegradability: with carbon as its structural base and titanium as an inert metal to life, Ti_3_C_2_T_*x*_ is considered nontoxic and biodegradable, making it highly promising for biomedical applications.^63,68^ These features position Ti_3_C_2_T_*x*_ as one of the most promising MXene for a wide range of applications, from electronic devices to the biomedical field. Furthermore, other studies have investigated different types of MXene as electrodes for supercapacitors. In the work by Bai et al.,^69^ the energy storage properties of Nb_*n*+1_C_*n*_T_2_ (*n* = 1 or 2) were analyzed, with and without the adsorption of Li atoms, using DFT principles. This study was based on experimental findings that identified Nb_*n*+1_C_*n*_T_2_ as a promising material for lithium-ion batteries.^70^ The authors employed pseudopotential methods with the generalized gradient approximation (GGA) and plane waves. The results demonstrated that Nb_2_C and Nb_3_C_2_ systems exhibit high theoretical capacitances, highlighting their potential for energy storage devices. It was found that surface terminations, such as –F and –O, significantly affect energy storage properties. These terminations promote charge transfer between the adsorbed atom and the surface but also reduce electronic conductivity and rate performance during charge and discharge processes. For the Nb_2_CLi_6_ system, a capacitance of 1981.56 F/cm^3^ was calculated. For systems with –F and –O terminations, Nb_2_CF_2_Li_6_ and Nb_2_CO_2_Li_6_, the capacitances were 1405.62 F/cm^3^ and 1617.22 F/cm^3^, respectively. Similarly, for Nb_3_C_2_Li_6_, Nb_3_C_2_F_2_Li_6_, and Nb_3_C_2_O_2_Li_6_, the theoretical capacitances were 2007.75 F/cm^3^, 1870.37 F/cm^3^, and 1710.40 F/cm^3^, respectively.

Xu et al.^71^ investigated the electronic properties and quantum capacitance (*Q*_C_) of Zr_2_CO_2_ MXene doped with Si, Ge, Sn, N, B, S, and F atoms using DFT-based computational calculations. The results showed that doping surface atoms significantly alters the material’s properties. For pristine Zr_2_CO_2_, a maximum *Q*_C_ of 407 μF/cm^2^ at a potential of −0.6 V was found, with a value of 32.3 μF/cm^2^ at 0 V. Introducing vacancies significantly improved the capacitance, especially under positive polarization, reaching up to 1938.4 μF/cm^2^ at 0 V. Among the dopants, B in the cathode raised the maximum capacitance to 1993 μF/cm^2^, while dopants such as F, Ge, N, S, Si, and Sn in the anode significantly increased capacitance. The standout result was with S doping, achieving a capacitance of 3293.7 μF/cm^2^ at 0.4 V. Simulations revealed that the enhanced capacitance is attributed to an increased density of states near the Fermi level. These findings suggest that atom doping in MXene, particularly Zr_2_CO_2_ derivatives, can significantly enhance electrode performance in SCs, greatly improving their energy storage capacity. Table 1 summarizes all the computational results discussed in this study.

**Table 1:** Summary of the Computational Works Explored in This Review^a^

electrode	electrolyte	method	capacitance	window voltage	ref.
Ti_3_C_2_O_2_	[HEMIm][NTf_2_]	MD	8.19 μF/cm^2^	2 V	^45^
Ti_3_C_2_(OH)_2_			11.56 μF/cm^2^		
Ti_3_C_2_(OH)_2_	[EMIM][PF_6_]	MD			^48^
Ti_2_CT_*x*_ (*T* = –O, –OH, –F)	aqueous	DFT	291.5 F/g		^51^
			252.2 F/g		
TiO_2_		DFTMD	0.4 F/m^2^		^53^
			0.3 F/m^2^		
Ti_3_C_2_T_*x*_	[EMIM][TFSI]	MD			^54^
graphene	[EMIM][Cl]	MD			^55^
Ti_3_C_2_T_*x*_	[EMIM][BF_4_]				
	[EMIM][OTF]				
	[EMIM][FSI]				
	[EMIM][TFSI]				
Ti_3_C_2_F_2_	[HEMIm][NTf_2_]	MD	26.5 to 40.6 F/g	3 V	^57^
	[EMIM][PF_6_]				
MXene	H_2_SO_4_	MD			^58^
MXene/graphene					
Ti_3_C_2_(OH)_2_	Li-TFSI/AN	MD			^61^
Nb_*n*+1_C_*n*_T_2_Li_6_	aqueous and organic	DFT	1405.62 to 2007.75 μF/cm^2^	0.5–0.8 V (aqueous)	^69^
				2–2.5 V (organic)	
Zr_2_CO_2_Si		DFT	407 to 3293 μF/cm^2^	–0.6 to 0.6 V	^71^
Zr_2_CO_2_Ge					
Zr_2_CO_2_Sn					
Zr_2_CO_2_N					
Zr_2_CO_2_B					
Zr_2_CO_2_S					
Zr_2_CO_2_F					

a Electrodes, electrolytes, simulation methods, capacitance, voltage window and respective references are shown.

### 3.2 – Experimental Results

Following the same perspective as the computational study by Sun et al.,^58^ the literature features several experimental studies highlighting the potential of hybrid electrodes in energy storage devices, as demonstrated in refs 72–75 . All these studies employed H_2_SO_4_ electrolytes in various concentrations to evaluate device performance. In the work by Yan et al.,^72^ high gravimetric and volumetric capacitances of 335 F/g and 1040 F/cm^3^, respectively, were achieved, with a performance retention of 61% at 1 V/s. In the study by Fan et al.,^58,73^ a retention rate of 69% at 500 mV/s was observed, with capacitances of 438 F/g and 1445 F/cm^3^. Zhou et al.^58,74^ and Yang et al.^58,75^ reported gravimetric capacitances of 140 F/g and 393 F/cm^3^, with performance retention rates of 50% at 10 A/g and 33% at 10 V/s, respectively. These results highlight the significant potential of hybrid electrodes, such as MXene/graphene, in energy storage systems. Both computational and experimental studies have demonstrated the excellent performance of these devices, establishing them as a promising approach for energy storage technology applications.

Yang et al.^76^ developed a rechargeable Zn@MXene capacitor based on Ti_3_C_2_, which is fully degradable and exhibits superior antiself-discharge functionality. The Zn@Ti_3_C_2_ cathode was synthesized using a constant-voltage deposition technology in a two-electrode system. The gel electrolyte was prepared from a ZnO_4_ solution and gelatin, resulting in a functional and environmentally friendly capacitor. The electrodes were fabricated through a method combining hydrofluoric acid synthesis and zinc atom deposition. The capacitor demonstrated high stability, retaining approximately 82.5% of its capacitance after 1000 charge–discharge cycles. Furthermore, it achieved a capacitance retention of 91.6% (302 F/cm^3^) at a rate of 3 A/g and an ultralow self-discharge rate of only 6.4 mV/h, significantly better than most previous supercapacitors, which exhibited rates exceeding 300 mV/h. Notably, the device showcased complete degradability. When exposed to a phosphate-buffered saline (PBS) solution containing hydrogen peroxide, the capacitor fully degraded within 8 days highlighting the potential of this device as a sustainable and efficient solution for energy storage applications.

In the study conducted by Vigneshwaran et al.,^77^ a novel low-cost hybrid electrode composed of CoMn_2_O_4_ incorporated with V_2_C was proposed for applications in energy storage devices. The synthesis of the MXene V_2_C was performed using a direct chemical process protocol starting from the MAX phase V_2_AlC. Initially, 5 g of the MAX phase were added to 100 mL of 50 wt % hydrofluoric acid and stirred using a magnetic stirrer operating at 300 rpm. The entire chemical process was carried out at approximately 27 °C for 92 h. After the reaction, the product was precipitated using centrifugation at 3500 rpm for 5 min per cycle and then washed with deionized water to remove impurities. Following the synthesis of V_2_C, the hybrid compound V_2_C@CoMn_2_O_4_ was prepared by gradually adding 100 mg of CoMn_2_O_4_ dropwise to 30 mg of V_2_C. Subsequently, 50 mL of 0.5 wt % cetyltrimethylammonium bromide (CTAB) solution were placed in a beaker and mixed overnight. The final V_2_C@CoMn_2_O_4_ composite was then isolated, as shown in Figure 9.

**Figure 9:**
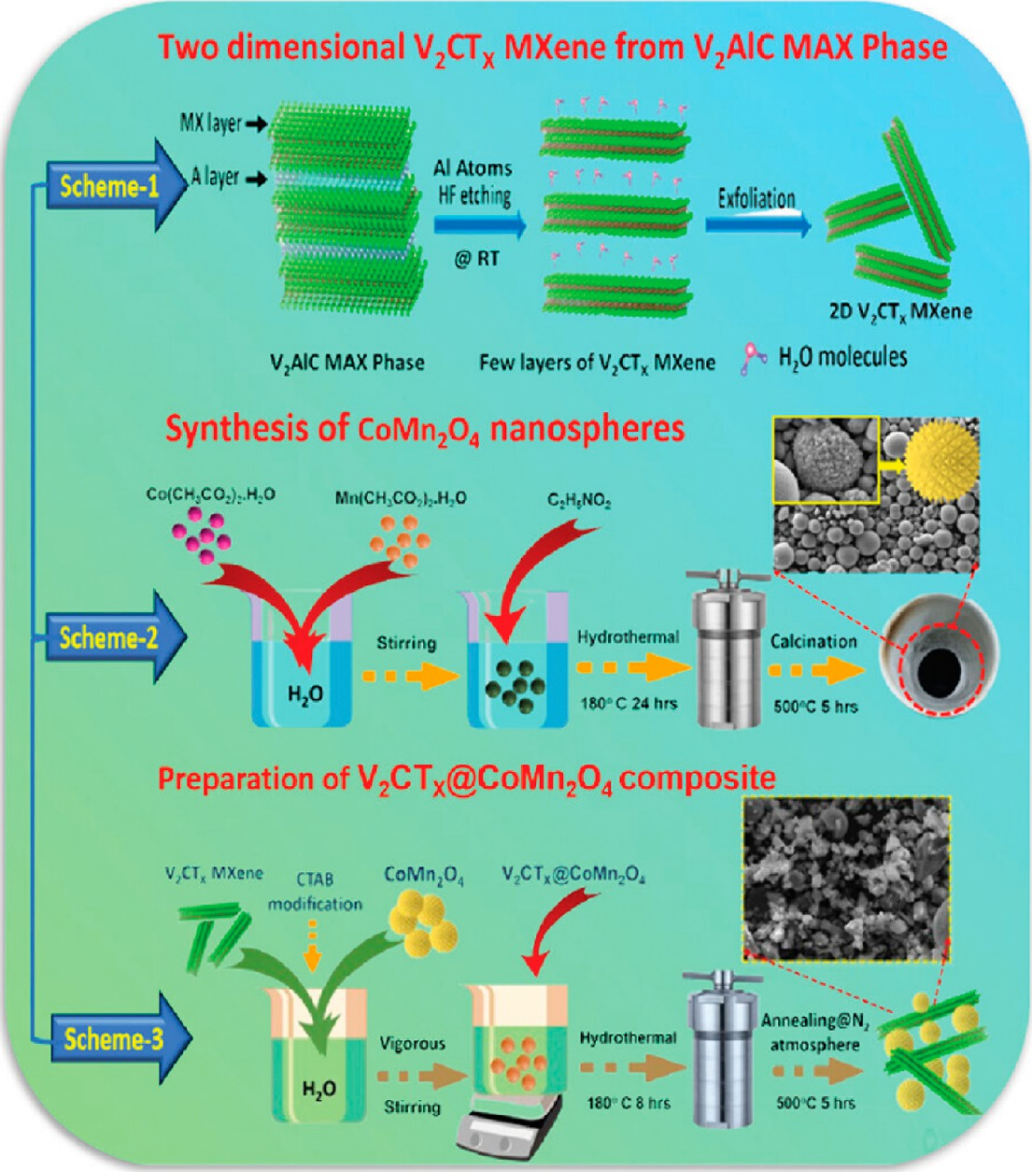
Schematic representation of the synthesis of the V_2_CT_*x*_@CoMn_2_O_4_. Reproduced with permission from Vigneshwaran et al.^77^ Copyright © 2024 American Chemical Society.

The authors investigated the electrochemical behavior of SCs using the hybrid electrode V_2_C@CoMn_2_O_4_ with a 1 M H_2_SO_4_ electrolyte. The analyses were conducted within a potential window of −0.2 to 0.7 V, with scan rates ranging from 10 to 100 mV/s. The results revealed a specific capacitance of 570 F/g at a current density of 1 A/g. Furthermore, the device exhibited an efficiency of approximately 96.5% after 10,000 charge–discharge cycles, highlighting the significant potential of the V_2_C@CoMn_2_O_4_ combination for energy storage applications.

Ai et al.^78^ prepared the V_2_C MXene using a mixture of HCl and NaF to remove Al atoms from the MAX phase V_2_AlC. Specifically, 1 g of V_2_AlC was added to a solution containing 50 mL of HCl and 2 g of NaF. Following the synthesis, electrochemical analyses were conducted on a supercapacitor composed of V_2_C electrodes and 1 M Na_2_SO_4_, H_2_SO_4_ and KOH electrolytes. The studies were performed within a voltage window of −0.4 to 0.4 V. For the V_2_C and Na_2_SO_4_ combination, a specific capacitance of 556.7 F/g was calculated at a scan rate of 2 mV/s. For H_2_SO_4_ and KOH electrolytes at the same rate (2 mV/s), specific capacitances of 113.4 F/g and 96.4 F/g, respectively, were obtained. Additionally, the V_2_C and Na_2_SO_4_ combination achieved a specific capacitance of 361 F/g at a scan rate of 10 mV/s. The results were compared with other studies that examined V_2_C-based electrodes synthesized using different methods.^79–82^ The findings demonstrated excellent performance, especially considering that the material preparation avoided the conventional use of HF, highlighting the efficiency of the alternative methodology employed. All results are summarized in Table 2.

**Table 2:** Summary of the Experimental Results Discussed in This Review, Showcasing the Electrode Materials, Electrolytes, Capacitance Values, Voltage Windows, Rate Performance, and Corresponding References, Providing a Detailed Comparison of Methodologies and Performance Metrics

electrode	electrolyte	capacitance	window voltage	rate performance	ref.
MXene/rGO	3 M H_2_SO_4_	335 F/g to1040 F/cm^3^		61% at 1 V/s	^58,72^
MXene/HGO	3 M H_2_SO_4_	438 F/g and1445 F/cm^3^		69% at 500 mV/s	^58,73^
MXene/rGO	1 M H_2_SO_4_	140 F/g and 393 F/cm^3^		50% at 10 A/g	^58,74^
PMG	3 M H_2_SO_4_	393 F/g		33% at 10 V/s	^58,75^
Zn/Ti_3_C_2_	ZnSO_4_/gelatin	302 F/cm^3^		91.6% at 3 A/g	^76^
V_2_C@CoMn_2_O_4_	1 M H_2_SO_4_	570 F/g	–0.2 to 0.7 V	96.5% over 10,000 cycles	^77^
V_2_C (NaF + HCl)	Na_2_SO_4_	556.7 F/g (2 mV/s)	–0.4 to 0.4 V		^78^
V_2_C (NaF + HCl)	H_2_SO_4_	113.4 F/g (2 mV/s)	–0.4 to 0.4 V		^78^
V_2_C (NaF + HCl)	KOH	96.4 F/g (2 mV/s)	–0.4 to 0.4 V		^78^
V_2_C (NaF + HCl)	Na_2_SO_4_	361 F/g (10 mV/s)	–0.4 to 0.4 V		^78^
V_2_C (KF + HCl)	Na_2_SO_4_	164 F/g (2 mV/s)			^78,79^
V_2_C (49 wt % HCl)	Mg_2_SO_4_	225 F/g (2 mV/s)			^78,80^
V_2_C (49 wt % HCl)	H_2_SO_4_	487 F/g (2 mV/s)			^78,80^
V_2_C (50 wt % HCl)	NaPF_6_	100 F/g (0.2 mV/s)			^78,81^
V_2_C (50 wt % HCl)	Na_2_SO_4_	120 F/g (10 mV/s)			^78,82^
V_4_C_3_T_*x*_ (40 wt % HCl)	1 M H_2_SO_4_	209 F/g (2 mV/s)	–0.25 to 0.2 V	97.2% over 10,000 cycles	^83^
V_4_C_3_T_*x*_ (40 wt % HCl)	1 M H_2_SO_4_	199 F/g (5 mV/s)	–0.25 to 0.2 V	97.2% over 10,000 cycles	^83^
V_4_C_3_T_*x*_ (40 wt % HCl)	1 M H_2_SO_4_	190 F/g (10 mV/s)	–0.25 to 0.2 V	97.2% over 10,000 cycles	^83^
V_4_C_3_T_*x*_ (40 wt % HCl)	1 M H_2_SO_4_	178 F/g (20 mV/s)	–0.25 to 0.2 V	97.2% over 10,000 cycles	^83^
V_4_C_3_T_*x*_ (40 wt % HCl)	1 M H_2_SO_4_	155 F/g (50 mV/s)	–0.25 to 0.2 V	97.2% over 10,000 cycles	^83^
V_4_C_3_T_*x*_ (40 wt % HCl)	1 M H_2_SO_4_	130 F/g (100 mV/s)	–0.25 to 0.2 V	97.2% over 10,000 cycles	^83^
Ti_3_C_2_/dodecaboratte	1 M H_2_SO_4_	366 F/g (2 mV/s)		70.4% over 5000 cycles	^89^
Ti_3_C_2_T_*x*_/Ag_2_CrO_4_	1 M KOH	75 F/g (20 mV/s)	–0.2 to 0.6 V		^90^
Ti_3_C_2_T_*x*_/Ag_2_CrO_4_	1 M KOH	40 F/g (40 mV/s)	–0.2 to 0.6 V		^90^
Ti_3_C_2_T_*x*_/Ag_2_CrO_4_	1 M KOH	29 F/g (70 mV/s)	–0.2 to 0.6 V		^90^
Ti_3_C_2_T_*x*_/Ag_2_CrO_4_	1 M KOH	28 F/g (80 mV/s)	–0.2 to 0.6 V		^90^
Ti_3_C_2_T_*x*_/Ag_2_CrO_4_	1 M KOH	26 F/g (100 mV/s)	–0.2 to 0.6 V		^90^
Ti_3_C_2_T_*x*_/Ag_2_CrO_4_	1 M H_2_SO_4_	525 F/g (10 mV/s)	–0.2 to 0.6 V		^90^
Ti_3_C_2_T_*x*_/Ag_2_CrO_4_	1 M H_2_SO_4_	348 F/g (20 mV/s)	–0.2 to 0.6 V		^90^
Ti_3_C_2_T_*x*_/Ag_2_CrO_4_	1 M H_2_SO_4_	239 F/g (40 mV/s)	–0.2 to 0.6 V		^90^
Ti_3_C_2_T_*x*_/Ag_2_CrO_4_	1 M H_2_SO_4_	176 F/g (70 mV/s)	–0.2 to 0.6 V		^90^
Ti_3_C_2_T_*x*_/Ag_2_CrO_4_	1 M H_2_SO_4_	161 F/g (80 mV/s)	–0.2 to 0.6 V		^90^
Ti_3_C_2_T_*x*_/Ag_2_CrO_4_	1 M H_2_SO_4_	148 F/g (100 mV/s)	–0.2 to 0.6 V		^90^
Ti_3_C_2_T_*x*_ aerogels	3 M H_2_SO_4_	438 F/g (10 mV/s)			^90,94^
Ti_3_C_2_T_*x*_ ion gels	ionic liquid	70 F/g (20 mV/s)			^90,95^
Ti_3_C_2_T_*x*_/PPy	1 M H_2_SO_4_	416 F/g (5 mV/s)			^90,96^
Ti_3_C_2_T_*x*_/PPy nanoparticles	1 M Na_2_SO_4_	184.36 F/g (2 mV/s)			^90,97^
Ti_3_C_2_T_*x*_ PMF	3 M H_3_PO_4_	218 F/g (2 mV/s)	0–1.4 V	82.42% over 4000 cycles	^98^
Ti_3_C_2_T_*x*_ PMF	3 M H_3_PO_4_	43 F/g (200 mV/s)	0–1.4 V	82.42% over 4000 cycles	^98^
Ti_3_C_2_T_*x*_ OMF	3 M H_3_PO_4_	297 F/g (2 mV/s)	0–1.4 V	88.6% over 10,000 cycles	^98^
Ti_3_C_2_T_*x*_ OMF	3 M H_3_PO_4_	108 F/g (200 mV/s)	0–1.4 V	88.6% over 10,000 cycles	^98^
Nb_2_CT_*x*_	1 M H_2_SO_4_	186 F/g (2 mV/s)	–0.35 to 0.2 V		^101^
Nb_2_CT_*x*_/CNT	1 M H_2_SO_4_	202 F/g (2 mV/s)	–0.35 to 0.2 V	80.3% over 5000 cycles	^101^
Ti_3_C_2_T_*x*_/SCNT	1 M KOH	129 F/g (2 mV/s)			^102^
Ti_3_C_2_T_*x*_ layered	1 M H_2_SO_4_	200 F/g (2 mV/s)			^103^

Wang et al.^83^ demonstrated that, as an electrode in supercapacitors, the MXene V_4_C_3_T_*x*_ exhibits excellent electrochemical performance using 1 M H_2_SO_4_ as the electrolyte. The MXene was synthesized via the conventional method, immersing the precursor MAX phase V_4_AlC_3_ in 40 wt % HF. To evaluate the electrode’s performance in the supercapacitor, different scan rates (2, 5, 10, 20, 50, and 100 mV/s) were tested within a potential window of −0.25 to 0.2 V. The results showed high specific capacitances of 209, 199, 190, 178, 155, and 130 F/g, respectively, for each scan rate. Additionally, the electrode exhibited higher capacitance at a scan rate of 2 mV/s compared to other studies using conventional MXene, such as Ti_3_C_2_T_*x*_ (117 F/g),^84^ Ti_3_C_2_/TiO_2_ (143 F/g),^85^ MoO_3_/Ti_3_C_2_T_*x*_ (151 F/g),^86^ Ti_3_C_2_T_*x*_/PVA (167 F/g),^87^ and N-doped Ti_3_C_2_T_*x*_ (192 F/g).^88^ In addition to the high capacitances, the authors highlighted the device’s excellent cycling stability, retaining 97.23% of its initial capacitance after 10,000 charge–discharge cycles at a current density of 10 A/g. These findings underscore the potential of V_4_C_3_T_*x*_ as a promising material for supercapacitor applications.

Li et al.^89^ highlighted a compound known as dodecaborate in their study, which has garnered attention due to its icosahedral structure formed by strong bonds between boron atoms. The innovative study proposed the use of a hybrid MXene/dodecaborate material in supercapacitors. The authors modified the MXene surface through ultrasonic treatment, adding ammonium and dodecaborate ions to the internal MXene surface via electrostatic adsorption. The choice of dodecaborate was primarily motivated by its considerable size and highly delocalized π bonding structure. Compared to smaller ions like SO_4_^–^, dodecaborate exhibits lower affinity for binding with cations, allowing cations in the electrolyte to move more freely and facilitating ion diffusion between MXene layers. This can significantly enhance capacitance and device efficiency. In the study, Ti_3_AlC_2_ MAX was synthesized using 1 g of Ti_3_AlC_2_ with 20 mL of a 40 wt % HF solution. For the preparation of the MXene/dodecaborate hybrid, 100 g of MXene and 20 mg of (NH_4_)_2_CO_3_ were added to 25 mL of deionized water. The electrochemical properties of the device were investigated using an aqueous 1 M H_2_SO_4_ solution as the electrolyte. The results showed a specific capacitance of 366 F/g at a scan rate of 2 mV/s, eight times higher than that of pure MXene (43 F/g) at the same rate. Furthermore, the MXene/dodecaborate hybrid electrode retained 70.4% of its performance after 5000 cycles, compared to only 49.3% for pure MXene. These findings demonstrate a substantial improvement in supercapacitor performance with dodecaborate incorporation, highlighting the potential of this approach.

Following the same concept of composite materials for electrodes, Yaqoob et al.,^90^ inspired by studies that utilized silver nanoparticles as promising tools for energy storage devices,^91–93^ proposed an electrode based on Ti_3_C_2_T_*x*_/Ag_2_CrO_4_. To synthesize the Ag_2_CrO_4_ nanoparticles, the sol–gel method was applied. In this synthesis approach, 4 g of silver nitrate and 3 g of sodium nitrate solution were prepared in 50 mL of deionized water, following the reactions

$$Ag\to Ag^{+}+e^{-}$$

$$2Ag^{+}+CrO_{4}^{2-}\to Ag_{2}CrO_{4}$$

Conversely, the conventional method was used to synthesize the Ti_3_C_2_T_*x*_ MXene, employing 200 mL of 39 wt % HF solution on the MAX phase. After synthesizing the MXene, the coprecipitation method was applied to prepare the composite electrode. Electrochemical investigations were performed using cyclic voltammetry, varying the electrode potential within a voltage window of −0.2 to 0.6 V. The electrolytes tested were 0.1 M H_2_SO_4_ and 1 M KOH, evaluated at various scan rates (10, 20, 40, 70, 80, and 100 mV/s). The results showed that, even at a low concentration of H_2_SO_4_, the capacitances were significantly higher than those observed with the KOH-based electrolyte. It was noted that the scan rate and specific capacitance are inversely proportional. The highest capacitance value found in the study was 525 F/g at a scan rate of 10 mV/s for the H_2_SO_4_ electrolyte. At the highest scan rate of 100 mV/s, the specific capacitance was 148 F/g for 0.1 M H_2_SO_4_, while the 1 M KOH solution yielded a considerably lower value of 26 F/g. In addition to these significant findings, the authors compared their data with other studies,^94–97^ demonstrating that the chosen combination of electrode and electrolyte offers superior capacitance compared to other models. These results underscore the potential of composite materials as electrodes for use in supercapacitors.

Li et al.^98^ explored a reaction between ethanol and phosphoric acid to expand the interlayer spacing of Ti_3_C_2_T_*x*_ MXene. In the process, ethanol and phosphoric acid intercalated between the MXene layers react under heating conditions, forming larger phosphate structures, which increase the interlayer spacing. This expansion not only enhances the charge/discharge rate but also improves the gravimetric and volumetric capacitances of the device, optimizing its electrochemical performance. Figure 10 shows the pure MXene (PMF) and the optimized MXene (OMF) after the reaction.

**Figure 10:**
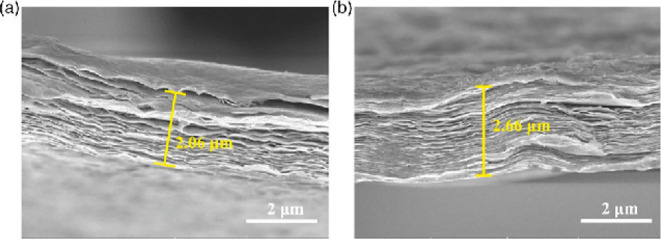
Cross-sectional scanning electron microscopy (SEM) images of (a) pristine MXene film (PMF) and (b) oxidized MXene film (OMF), highlighting morphological differences after the oxidation process. Reprinted with permission under a Creative Commons (CC BY 4.0), from ref 98. Copyright 2022 John Wiley & Sons.

The preparation of the Ti_3_C_2_T_*x*_ MXene colloidal solution began with the etching of 1 g of Ti_3_AlC_2_ precursor in a mixture of 20 mL of 9 M HCl and 1.6 g of LiF at 50 °C for 30 h. The resulting product was washed with deionized water (DI) through repeated centrifugation at 3500 rpm for 5 min until the pH of the supernatant reached 6. The sediment was then redispersed in DI water, sonicated for 1 h with ice cooling and an Ar flow to prevent oxidation, and centrifuged again at 3500 rpm for 1 h to separate the delaminated MXene colloidal solution, with the supernatant collected. To prepare a freestanding MXene film optimized for ion diffusion, 7 mL of an aqueous MXene solution (1.8 mg/mL) was vacuum filtered using a Celgard 3501 membrane, forming a densely stacked film. After drying overnight in a desiccator, the film was treated in a mixture of 50 mL ethanol and 400 mg phosphoric acid at 60 °C for 6 h to expand the interlayer spacing. The film was then washed with acetone and ethanol and dried at room temperature, resulting in a material optimized for ion diffusion. The results showed that, for the optimized MXene, the gravimetric capacitance was 297 F/g at a scan rate of 2 mV/s with a 3 M H_3_PO_4_ electrolyte. At a higher scan rate of 200 mV/s, the capacitance was 108 F/g, significantly outperforming the nonoptimized MXene. For the unoptimized MXene, the gravimetric capacitance was 218 F/g at 2 mV/s and only 43 F/g at 200 mV/s.

MXene using Nb as the transition metal also show great promise for energy storage devices,^99^ despite having lower electrical conductivity compared to Ti-based MXene.^100^ Xiao et al.^101^ proposed an innovative strategy for synthesizing crystalline-phase Nb_2_CT_*x*_ and demonstrated that the electrochemical performance of this material can be significantly enhanced by adding carbon nanotubes (CNTs) to the aqueous electrolyte, improving conductivity and the overall performance of energy storage devices. In their study, a 1 M H_2_SO_4_ solution was used as the electrolyte, with cyclic voltammetry and galvanostatic charge–discharge processes conducted within a potential window of −0.35 to 0.2 V. At a scan rate of 2 mV/s, the authors reported specific capacitances of 186 F/g for pure Nb_2_CT_*x*_ electrodes and 202 F/g for Nb_2_CT_*x*_/CNT composite electrodes. For comparison, Fu et al.^102^ investigated a combination of Ti_3_C_2_T_*x*_ with single-walled carbon nanotubes (SCNTs) in a 1 M KOH electrolyte and found a specific capacitance of approximately 129 F/g at a scan rate of 2 mV/s. Hu et al.^103^ used the same electrolyte as in the study by Xiao et al.,^101^ a 1 M H_2_SO_4_ solution, and achieved similar capacitance values. At the same scan rate of 2 mV/s, they reported a specific capacitance of approximately 200 F/g, further highlighting the potential of the electrolyte and material for energy storage applications.

### 3.3 Comparison between Computational and Experimental Results

A significant variation in capacitance values is observed when comparing computational and experimental data on the performance of MXene-based materials in supercapacitors. This discrepancy reflects both the diversity of simulated conditions and the inherent challenges in replicating experimental scenarios within theoretical models.

In computational studies, for instance, the theoretical volumetric capacitance of sulfur-doped Zr_2_CO_2_ reached values up to 3293 μF/cm^2^.^71^ Similarly, the predicted gravimetric capacitances for Ti_2_CO_2_ and Ti_2_CF_2_ were 291.5 F/g and 252.2 F/g, respectively.^51^ In contrast, experimental data show a wide range of values, from 26 F/g (Ti_3_C_2_T_*x*_/Ag_2_CrO_4_ electrode)^90^ to 570 F/g for a hybrid V_2_C@CoMn_2_O_4_ electrode,^77^ and down to 556.7 F/g for V_2_C in Na_2_SO_4_.^78^ These variations indicate that experimental results sometimes surpass theoretical predictions, highlighting the complexity involved in accurately modeling real systems.

Discrepancies between computational and experimental outcomes can be attributed to several factors. First, theoretical models often assume ideal conditions, such as perfectly crystalline structures, the absence of defects, and a uniform distribution of functional groups. In real samples, however, factors like impurities, morphological variations, functionalization heterogeneity, and limitations in electrode deposition methods can significantly impact electrochemical performance.

Additionally, simulations based on DFT or MD operate on restricted time and spatial scales, which do not fully capture effects related to cyclic degradation, macroscopic ion transport, or heat accumulation and dissipation. For example, while simulations suggest that −OH terminations favor cation orientation and increase capacitance,^45^ this behavior does not always translate directly into real devices due to chemical instability or competition with other species present in electrolytes.

Another critical aspect is the choice of electrolyte. Many theoretical studies use ionic liquids like [EMIM]­[TFSI] or [HEMIm]­[NTf_2_], while experimental investigations often rely on aqueous solutions, such as H_2_SO_4_ or Na_2_SO_4_, which have very different physicochemical properties. This difference directly affects the structure of the EDL and, consequently, the measured capacitance.

Therefore, although theoretical and experimental results may not coincide in magnitude, they are complementary. Simulations allow for the systematic exploration of variables and a deeper understanding of fundamental charge storage mechanisms. In turn, experiments validate and challenge these predictions, contributing to the development of materials with greater efficiency and real-world applicability in commercial devices.

### 3.4 Carbon-Based Electrodes versus MXene Electrodes

Energy storage in SCs is directly related to the accumulation of charges at the electrode–electrolyte interface, driven by electrostatic interactions between their components^104^ or with the electron transfer due to redox reactions between electrode and electrolyte.^7^ Consequently, the electrical properties of the device, particularly its capacitance, are highly dependent on the surface area and the material used for the electrode. In this regard, carbon-based materials are commonly used in EDLCs due to their high surface area, excellent electrical conductivity, and remarkable chemical stability. Numerous studies in the literature, both computational and experimental, describe the application of these materials and their efficiency in enhancing the electrochemical performance of supercapacitors.

Indeed, various types of carbon-based materials exist with unique properties, among which carbon nanotubes (CNTs), that models can be seen in Figure 11 stand out.^105^ Discovered in 1991 by Iijima,^106^ CNTs have been extensively studied as potential candidates for supercapacitors due to their high surface area, exceptional electrical conductivity, and chemical stability. Niu et al.,^107^ for instance, investigated the electrochemical performance of CNTs and identified high specific capacitances ranging from 13 to 113 F/g across different frequencies, highlighting the great potential of these materials for energy storage applications.

**Figure 11:**
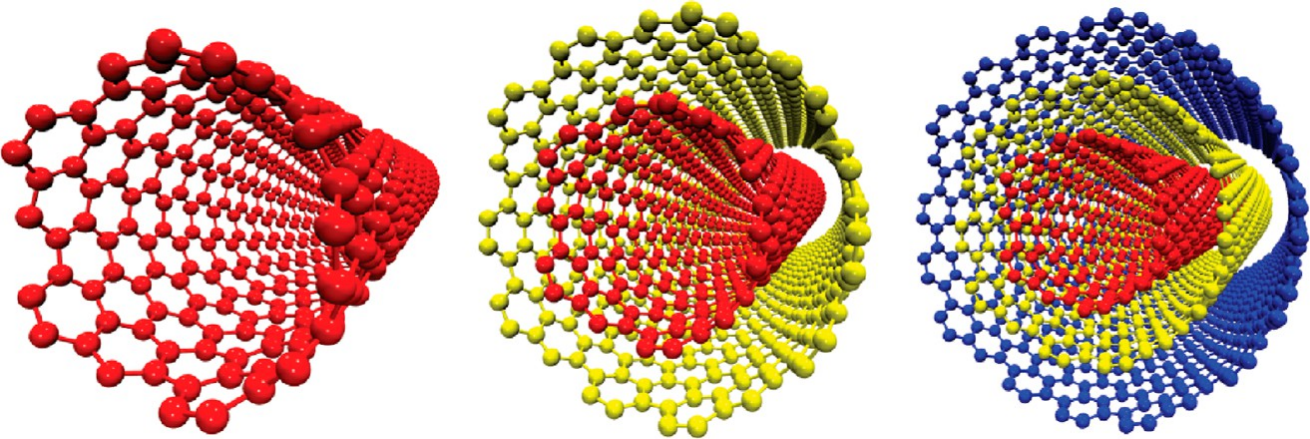
Surface and Internal view of a single and multiwall carbon nanotube. Adapted with permission from Rathinavel et al.^105^ Copyright © 2021 Elsevier.

Easy chemical modification makes CNTs highly versatile for electrochemical applications. In ref 108 it was shown that relatively simple modifications can significantly enhance the performance of CNTs as electrodes in supercapacitors. In that study, the authors employed MD simulations to assess how electric potential, electrode curvature, and temperature influence the capacitance of CNT-based supercapacitors using [EMIM]­[Tf_2_N] as the electrolyte. The results indicated that the capacitance varied by only 2.5% when temperature increased from 260 to 400 K. Furthermore, the electrode curvature was found to play a significant role in the capacitive behavior of the device, with capacitance increasing as the curvature decreased with capacitance magnitude ∼0.04–0.06 F/m^2^.

Chou et al.^109^ achieved 167.5 F g^–1^ at 77 mA g^–1^ and retained 88% capacitance after 3000 cycles. Similarly, Gueon et al.^110^ investigated MnO_2_ in the form of nanoflakes combined with CNTs. In this case, even higher capacitances of approximately 370 F/g were achieved at a current density of 0.5 A/g, with a power density of 810 J/g. Compared to studies using only CNTs, this combination demonstrated a 14-fold improvement in performance.

In addition to CNTs, activated carbon (AC)-based electrodes stand out due to their low production cost, high chemical stability, large porous surface area, and high volumetric capacitances.^111^ Low cost and high chemical stability make activated carbon (AC) a popular electrode material for supercapacitors. In ref 112 Shi et al. developed an electrochemical deposition method to prepare a Ni_3_S_2_/AC nanosheet composite, which exhibited high capacitances of 2797 F/g. Furthermore, the device showed high energy densities of 198 J/g and excellent cycle performance, retaining 83% of its initial capacitance after 10,000 charge–discharge cycles. Yan et al.^113^ reported that doping activated carbon with N, S, and O atoms significantly enhances the electrochemical performance of supercapacitors. The study demonstrated specific capacitances of approximately 357 F/g at a current density of 0.5 A/g. At higher current densities, around 50 A/g, capacitances of 262 F/g were achieved. Moreover, the material exhibited excellent cycle stability, with a capacitance retention of approximately 98% after 10,000 charge–discharge cycles at 2 A/g.

Similarly, Shang et al.,^114^ Charoensook et al.,^115^ and Guo et al.^116^ also reported high capacitances for doped AC electrodes. In ref 114 the researchers employed doping methods using N and S atoms with a 2 M KOH electrolyte. At a current density of 0.5 A/g, a capacitance of 285 F/g was achieved. In ref 115,116 higher KOH concentrations (6 M) were utilized, resulting in capacitances ranging from 324 to 332 F/g. While ref 114 focused on nitrogen doping, ref 116 investigated other dopants, such as phosphorus and sulfur. These findings demonstrate that, in addition to activated carbon being an excellent electrode material, the combination with dopant atoms significantly enhances device performance, making them even more efficient for supercapacitor applications.

Graphene, a carbon allotrope, is also noteworthy due to its unique properties. It is a two-dimensional material with sp^2^ hybridization that can be obtained through mechanical exfoliation of graphite or graphite oxides. Graphene stands out among other carbon compounds not only for its high porous surface area but also for its exceptional ionic conductivity, making it highly promising for energy storage applications. Moreover, combining multiple layers of graphene can be a viable strategy to enhance the performance of SCs. Numerous studies have highlighted the use of graphene as an electrode material in SCs. For instance, in ref 117 exfoliated graphene oxides were synthesized using simple chemical techniques from graphite, and the electrochemical performance of an SC was evaluated. The results showed a specific capacitance of approximately 146 F/g and a gravimetric energy density of 72 J/g.

In our previous works, we also evaluated the performance of EDLCs with graphene electrodes. In ref 18 we investigated the electrical and structural properties of EDLCs with graphene electrodes and amino-acid–based hydrated electrolytes, achieving capacitances ranging from 2.1 to 2.7 μF/cm^2^ and gravimetric energy densities of approximately 6.5 J/g.

In ref 118 we conducted a systematic study on the use of graphene and graphyne as electrode materials. The results indicated that the device’s performance is directly influenced by the electrode’s structure and the EDL composition. Simulations conducted within an electrochemical window of about 2.0 V revealed that graphyne electrodes exhibited significantly higher total capacitances compared to graphene electrodes, with percentage differences ranging from 3 to 14%. Furthermore, the study found that, in terms of dynamic properties, both graphene and graphyne electrodes had negligible impact on ionic mobility. Additionally, the potential for hybrid devices combining graphene and graphyne electrodes was highlighted, showing the possibility of further improving energy storage performance.

Vivekchand et al.^119^ investigated graphene prepared through three different methods as electrodes for electrochemical supercapacitors, achieving capacitances in the range of 100–205 F/g, highlighting the material’s potential for energy storage applications. In ref 120 both graphene and graphyne electrodes were evaluated through computational simulations. The results revealed capacitances of 2.48 μF/cm^2^ and 2.58 μF/cm^2^, respectively, for supercapacitors utilizing the ionic liquid [EMIM]­[BF_4_] and NaCl as electrolytes.

In ref 121 Silva et al. investigated the structural properties of supercapacitors based on graphene decorated with C60 fullerenes, using MD simulations to understand how different ionic liquids influence the electrochemical performance of the devices. The study revealed that supercapacitors with graphene decorated with C60 fullerenes achieved specific capacitances of up to 135 F/g, approximately 32% higher than devices with pure graphene. This enhancement was attributed to the increased porosity introduced by the fullerenes, which facilitates ion mobility and improves the overall performance of the device.

Indeed, with this theoretical framework, it becomes clear that both carbon-based materials and MXene demonstrate promising results in terms of capacitance and energy storage. However, it is noteworthy that these materials tend to be preferentially used in EDLCs and pseudocapacitors, respectively. Carbon materials primarily operate via electric double-layer capacitance, whereas MXene favor pseudocapacitance through redox reactions.

This distinction arises from the differing energy storage mechanisms. In EDLCs, storage relies solely on capacitive processes, based on the accumulation of charges near the electrode surfaces. Carbon materials, such as CNTs, ACs, and graphene, feature highly conductive surfaces and porous structures, ideal for maximizing capacitance through ion adsorption. Furthermore, carbon electrodes exhibit exceptional stability over a wide range of potentials, as they do not involve chemical reactions, leading to a longer device lifespan. Another key factor favoring the use of carbon materials in EDLCs is their low production cost and compatibility with various electrolytes, making them an economical and versatile choice for energy storage applications.

Conversely, MXene feature highly reactive atomic layers in their structure, which facilitate redox reactions. These chemical processes enable MXene-based pseudocapacitors to achieve higher capacitances than EDLCs, provided that the electrolyte is appropriately chosen. Additionally, MXene exhibit exceptional electrical conductivity due to the presence of transition metals in their composition, allowing for rapid charge transfer. Another noteworthy characteristic is the significantly higher volumetric density of MXene compared to carbon-based materials, making them ideal for devices requiring high charge density in compact volumes, such as electric or hybrid vehicles. Furthermore, the structure of MXene can be easily modified by incorporating T$_x$ terminations, which serve as active sites for chemical reactions, further enhancing the performance of pseudocapacitors. Table 3 summarizes these advantages and the comparisons between MXene and carbon-based materials.

**Table 3:** Properties Comparison: Carbon-Based Electrodes vs MXene for Supercapacitors Energy Storage

property	carbon-based electrodes (EDLCs)	MXene (pseudo capacitors)
storage mechanism	physical (electric double layer)	chemical (redox reactions)
specific capacitance	moderate	high
volumetric density	low	high
cycling stability	very high	good (degrades over long cycles)
electrical conductivity	moderate	excellent
cost	low	relatively high
functionalization	limited	surface termination groups
electrolyte compatibility	broad (aqueous, organic, ionic liquids)	broad

## 4 Current Challenges

Despite the significant advancements in MXene research, several challenges must be addressed for their widespread adoption, particularly in energy storage applications. One of the most critical issues lies in the synthesis process. Although MXene show remarkable potential, their large-scale production is hindered by the widespread use of hydrofluoric acid (HF)-based etching—a method that is both hazardous and environmentally damaging.^12,27^ The success of this synthesis is highly dependent on the M–A bond energy and parameters such as HF concentration, etching time, and reaction temperature. For example, increasing the HF concentration can accelerate the etching process, but it also risks damaging the lateral structure of the MXene, compromising material integrity.^28–30^ To overcome these limitations, several alternative synthesis routes have been explored, including alkali-based,^31–34^ electrochemical etching,^21,35,36^ molten salt,^40,41^ and hydrothermal methods.^42^ These approaches enable better control over surface terminations and structural characteristics while reducing environmental risks. However, further development of scalable, reproducible, and sustainable synthesis strategies remains essential for the commercial viability of MXene.

Another major challenge is related to the structural stability of MXene during device operation. Computational studies have revealed that the volumetric expansion and contraction of electrode layers, caused by ion oscillations during charging and discharging, may compromise the long-term durability and cyclic performance of these materials.^48,54,55^ This dynamic behavior, which results from interlayer ion movement and density fluctuations, demands more comprehensive theoretical and experimental investigations to fully understand its implications on electrode lifespan and device reliability.

Scalability remains a key bottleneck in translating laboratory-scale synthesis into industrial applications. While Ti-based MXene such as Ti_3_C_2_T_*x*_ are relatively easier to produce,^63,64^ many other compositions face significant synthesis challenges due to precursor limitations, harsher etching conditions, or lower yields.^29,30^ Moreover, achieving consistent control over flake size, thickness, and surface terminations on a large scale continues to be difficult, which affects the reproducibility and uniformity of MXene-based devices. Addressing these limitations requires not only optimizing synthesis protocols but also developing scalable postprocessing and integration techniques compatible with industrial manufacturing.

Surface terminal instability also represents a significant challenge. The functional groups introduced during synthesis (−F, −OH, −O, etc.) are highly sensitive to environmental conditions such as humidity, temperature, and pH.^16,17,27^ These terminations play a pivotal role in dictating the electronic structure, ion adsorption behavior, and overall electrochemical performance of MXene.^45,51,55^ Additionally, limitations in computational modeling methods must be considered. While the constant potential method (CPM) is a powerful tool for simulating metallic electrodes like MXene,^60^ it does not account for electronegativity differences between atoms, which can lead to inaccuracies in charge distribution predictions. To overcome this, Lin et al.^61^ introduced the heteroatomic constant potential method (HCPM), which incorporates atomic electronegativity and improves the realism of simulations involving heteroatomic electrodes. Although promising, HCPM still requires refinement, particularly through the development of more accurate interaction parameters, potentially via first-principles methods like DFT.

Finally, addressing the long-term sustainability of MXene is crucial for their broader implementation in energy storage technologies. This includes minimizing the environmental impact of their synthesis processes, developing strategies to replace hazardous or nonrenewable components with eco-friendly alternatives, and advancing recycling and recovery methods for end-of-life devices. Moreover, reducing reliance on scarce or toxic elements is essential to ensure the scalability, safety, and ecological compatibility of MXene-based systems. These efforts are fundamental to aligning the development of MXene with the principles of green chemistry and sustainable engineering.

## 5 Summary and Outlook

MXene, a novel family of two-dimensional materials, have recently turned themselves into one of the most emerging materials for energy storage applications, particularly in supercapacitors. Characterized by high electrical conductivity, tunable surface terminations, and swift ion transport, these materials represent a unique intersection of materials science domains. This review evidenced MXene as prospective electrodes for supercapacitors by emphasizing computational and experimental results that demonstrate their unique performance in electrochemical applications. In addition, their tunable properties to host various functional groups and to suit different electrolytes allows for further customization and offers potential applicability to a variety of energy storage technologies. These features elucidate the increasing significance of MXene in advancing supercapacitor efficiency and performance.

The development of MXene-based hybrid supercapacitors, which combine transition metal dichalcogenides (TMDs) with carbon-based materials (such as graphene and CNT) is one of such interesting pathways in MXene research. These hybrid systems exploit the high surface area, mechanical stability of carbon materials together with the outstanding electrical properties and ion accessibility of MXene. Such hybrids provide potential for high energy density in combination with power density by integrating the electric double-layer capacitance of the carbon materials with the pseudocapacitive behaviors of MXene. Experimental results have shown enhancements in charge storage and cycling stability, and it is believed that this class of materials will play an important role in future high-performance energy storage devices.

However, the large-scale promise of MXene is hampered by many issues, and their synthesis is one of the most important issues. Existing processes like HF-based etching, however, are frequently dangerous and damaging to the environment. Recent developments in synthesis methods (e.g., alkali, hydrothermal, molten salt) have demonstrated their potential to mitigate these challenges. These alternative routes provide more controlled surface termination and structural characteristics in MXene, thus alleviating environmental hazards. Advancing scalable and sustainable synthesis approaches is crucial to the commercialization of MXene and their widespread use.

In terms of future directions, an important aspect will be the synergetic hybridization of MXene with advanced carbonaceous materials. To achieve this, researchers ask for the design of hybrids where the complementary characteristics of materials are optimized to give devices that simultaneously enrich the electric double-layer and pseudocapacitive mechanisms. The ability of MXene to be custom functionalized on their surfaces along with the structural and conductive benefits of carbon materials will be the precursor of next-generation hybrid supercapacitors.

MXene have the potential to make seminal contributions across disciplines, but they must be developed in accordance with global sustainability goals as they continue to garner attention from the scientific community. This includes tackling the environmental consequence of their production as well as investigating biodegradable and renewable substitutions for linking materials and enhancing recycling systems. Additionally, decreasing reliance on rare or toxic components is crucial for the broader integration of MXene into sustainable energy storage applications. Addressing these issues, MXene-based systems have the ability to reshape the energy storage field, leading to a more environmentally friendly and sustainable future.

## 6 Conclusions

In conclusion, MXene represent a transformative advancement in energy storage materials, offering unmatched versatility and performance as supercapacitor electrodes. Their unique properties, including tunable functionalities and compatibility with hybrid systems, position them as a cornerstone for next-generation energy storage devices. While challenges such as scalable synthesis and environmental concerns remain, ongoing innovations in these areas pave the way for broader adoption. The integration of MXene with carbon-based materials, along with a focus on sustainability, holds immense potential to redefine the capabilities of supercapacitors. As research progresses, MXene are poised to play a crucial role in meeting the growing demand for efficient and sustainable energy storage solutions.
